# scMET: Bayesian modeling of DNA methylation heterogeneity at single-cell resolution

**DOI:** 10.1186/s13059-021-02329-8

**Published:** 2021-04-20

**Authors:** Chantriolnt-Andreas Kapourani, Ricard Argelaguet, Guido Sanguinetti, Catalina A. Vallejos

**Affiliations:** 1 MRC Institute of Genetics and Molecular Medicine, University of Edinburgh, Edinburgh, UK; 2 School of Informatics, University of Edinburgh, Edinburgh, UK; 3 European Bioinformatics Institute (EMBL-EBI), Hinxton, UK; 4 SISSA, International School of Advanced Studies, Trieste, Italy; 5 The Alan Turing Institute, London, UK

**Keywords:** DNA methylation, Single-cell, Epigenetic heterogeneity, Hierarchical Bayes

## Abstract

High-throughput single-cell measurements of DNA methylomes can quantify methylation heterogeneity and uncover its role in gene regulation. However, technical limitations and sparse coverage can preclude this task. scMET is a hierarchical Bayesian model which overcomes sparsity, sharing information across cells and genomic features to robustly quantify genuine biological heterogeneity. scMET can identify highly variable features that drive epigenetic heterogeneity, and perform differential methylation and variability analyses. We illustrate how scMET facilitates the characterization of epigenetically distinct cell populations and how it enables the formulation of novel hypotheses on the epigenetic regulation of gene expression. scMET is available at https://github.com/andreaskapou/scMET.

**Supplementary Information:**

The online version contains supplementary material available at (10.1186/s13059-021-02329-8).

## Background

DNA methylation (DNAm) at cytosine residues plays an important role in the regulation of gene expression [1]. It is also critical for a broad range of biological processes, including X-chromosome inactivation, genomic imprinting, and cancer [2–4]. The gold standard approach to profile DNAm at single-base resolution is to treat DNA with sodium bisulphite, which efficiently converts unmethylated cytosines to uracils, while leaving methylated cytosines unmodified [5]. Although bulk bisulphite sequencing (BS-seq) experiments have paved the way for mapping the methylome landscape across different tissues, they fall short of explaining the inter-cellular methylation heterogeneity and quantifying its dynamics in a variety of biological contexts [6].

More recently, advances in sequencing technologies have enabled the development of protocols that profile DNAm with single-cell resolution (e.g., scBS-seq, [7, 8]) and multiplexing protocols offer scalability to thousands of cells in a single experiment [9, 10]. In contrast to gene expression signatures from scRNA-seq experiments, which are influenced by the environment, DNA methylation profiles are highly distinct between cell types and stable across individuals and over the life span [11, 12]. Moreover, while scRNA-seq assays might fail to capture information about genes with moderate expression levels, cell-level measurements of DNAm offer a more complete coverage across genomic regions [9]. However, due to the small amounts of initial genomic DNA and the destructive nature of bisulphite on nucleic acids, the output data are often noisy and extremely sparse; that is, a large proportion of CpG dinucleotides is not observed (ranging from 80 to 95%). While tailored computational imputation methods such as Melissa [13] and DeepCpG [14] might ameliorate the sparsity problem, disentangling genuine epigenetic variability from technical biases remains a formidable problem.

Here, we present scMET, a Bayesian framework that addresses the statistical challenges associated with sparse scBS-seq data and provides novel functionality that is tailored to single-cell-level datasets. To overcome sparsity, scMET aggregates the input data within regions (hereafter also referred to as genomic *features*): either by combining CpG information in a sliding window approach or by using pre-annotated contexts, such as promoter regions or enhancers [7, 15]. To dissect genuine epigenetic variability from the many confounding technical biases, scMET adopts a hierarchical model specification which shares information across cells and genomic features, while incorporating feature-level characteristics (e.g., CpG density). Critically, scMET introduces residual *overdispersion* estimates as a measure of DNAm variability that is not confounded by mean methylation. These estimates can be used to perform differential DNAm variability testing among groups of cells, embracing the cellular resolution of the data to provide novel insights which are not possible using traditional differential mean tests on bulk data [16]. scMET can also identify highly variable features (HVFs) which, among others, can be used as input for unsupervised clustering analyses.

scMET scales readily to thousands of cells and features, making it a powerful tool for large-scale single-cell epigenetic studies. Our results both on simulated and real datasets demonstrate that it can accurately and robustly quantify DNAm heterogeneity. Results on two recent large-scale datasets show that scMET detects biologically relevant highly variable features which result in improved clustering performance. In addition, we show that scMET can facilitate the interrogation of single-cell multi-omics assays, yielding novel biological hypotheses on the role of epigenetic variability in gene regulation in early development.

## Results

***Quantifying cell-to-cell DNAm heterogeneity with scMET***

Standard statistical models for count data, such as the Poisson and binomial distributions, do not always capture the properties of data generated by high-throughout sequencing assays (e.g., RNA sequencing, bisulphite sequencing). In such cases, the data typically exhibit higher variance than what is predicted by these models — this is often referred to as *overdispersion* [17, 18]. This overdispersion may relate to *technical variation* (e.g., differences in sequencing depth) or to *biological variation* between the units of interest (e.g., cells or subjects) that is linked to genetic, environmental, or other factors. Disentangling these sources of variation is a major challenge in computational biology.

**Fig. 1:**
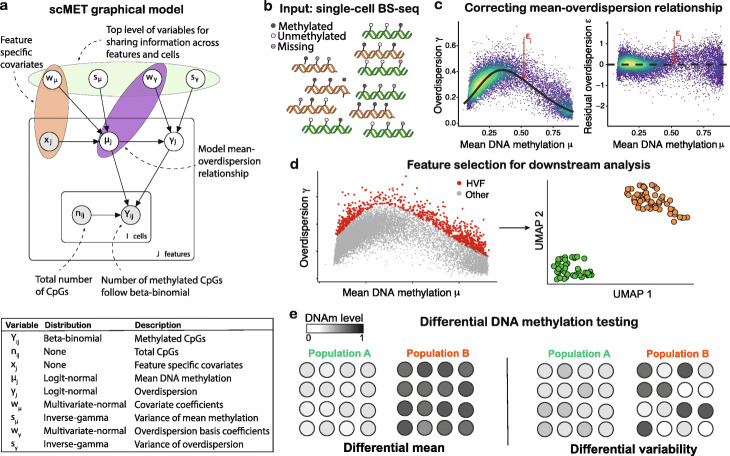
Graphical outline for scMET. **a** Overview of the scMET probabilistic graphical model. The random variables and data that form the model, along with the distributional assumptions, are shown. Input values are denoted by gray circles. Model parameters are denoted by white circles. **b** scMET uses single-cell DNAm data as input. The data could consist of measurements obtained from different groups of cells, such as experimental conditions or cell types (represented by green and orange colors in the diagram). For each region of interest (e.g., promoters), the input data is recorded in terms of the number of CpG sites for which a valid measurement was recorded and, among those, the number of methylated CpG sites. Note that many CpG sites will not be covered by a read (denoted by red color), leading to sparse information per genomic region. **c** By combining a hierarchical beta-binomial specification with a generalized linear model framework, scMET captures the mean-overdispersion relationship (left) that is typically observed in bisulphite sequencing readouts and derives residual overdispersion estimates that are not confounded by mean methylation (right). **d** scMET can be used to identify HVFs that drive epigenetic heterogeneity within a cell population. For example, these could be used as the input of dimensionality reduction techniques or clustering analyses. **e** scMET uses a probabilistic decision rule to perform differential methylation analysis: to identify features that show differences in mean methylation (left) and/or methylation variability (right) between pre-specified groups of cells

To disentangle technical from biological variability and overcome data sparsity, scMET couples a hierarchical beta-binomial (BB) model with a generalized linear model (GLM) framework (Fig. 1a, b). For each cell *i* and feature *j*, the input for scMET is the number of CpG sites that are observed to be methylated (*Y*_*ij*_) and the total number of sites for which methylation status was recorded (*n*_*ij*_), see Additional file 1: Figure S1. The BB model uses feature-specific mean parameters *μ*_*j*_ to quantify overall DNAm across all cells and biological *overdispersion* parameters *γ*_*j*_ as a proxy for cell-to-cell DNAm heterogeneity. The latter capture the amount of variability that is not explained by binomial sampling noise, which would only account for technical variation (see Additional file 1: Section S2.1). Hence, *γ*_*j*_ is akin to the overdispersion term used in negative binomial models for RNA-seq data (e.g., [19]). Although BB models have been developed for bulk DNAm data (e.g., [20, 21]), they typically use data from individual CpG sites as input, a strategy prone to fail for the highly sparse scBS-seq data.

The GLM framework is incorporated at two levels. Firstly, to introduce feature-specific covariates **x**_*j*_ (e.g., CpG density) that may explain differences in mean methylation *μ*_*j*_ across features. Secondly, similar to [22], we use a non-linear regression framework to capture the mean-overdispersion trend that is typically observed in high-throughput sequencing data, such as scBS-seq (Fig. 1c). Critically, this trend is used to derive *residual overdispersion* parameters *ε*_*j*_ — a measure of cell-to-cell variability that is not confounded by mean methylation. Feature-specific parameters are subsequently used for (i) feature selection, to identify highly variable features (HVFs) that drive cell-to-cell epigenetic heterogeneity (Fig. 1d), and (ii) differential methylation testing, to highlight features that show differences in DNAm mean or variability between specified groups of cells (Fig. 1e).

By using a Bayesian formulation, scMET infers the posterior distribution for all model parameters (Methods). Moreover, a variational Bayes scheme [23] permits scalable analysis to thousands of cells and features (Additional file 1: Figure S2), while having comparable posterior inference performance when compared to a Markov Chain Monte Carlo implementation (Additional file 1: Figure S3 and S4). As in [24], the output generated by scMET is used to implement a probabilistic decision rule to enable HVF selection and differential methylation testing. The decision rule is calibrated to control the expected false discovery rate (EFDR, [25]). A more detailed description of the model specification and its implementation is provided in the “Methods” section. scMET is implemented as an R package and is available at https://github.com/andreaskapou/scMET.

scMET can be combined with different choices for the input set of features. As a default, we recommend these to be defined by existing pre-annotated contexts (e.g., enhancers), as these can facilitate downstream interpretation. However, scMET can also be used for de novo annotation of regulatory regions by using sliding windows as input features (see the “Methods” section).

***Benchmarking scMET on synthetic data***

First, we benchmark the performance of scMET using synthetic data. To mimic the properties observed in real scBS-seq data, we simulated features with rich and poor CpG density (see the “Methods” section for details about the simulation settings). We compared mean and overdispersion estimates obtained by scMET with respect to BB maximum likelihood estimates (BB MLE), which were obtained separately for each feature. As expected, mean parameters *μ*_*j*_ are easier to infer and estimates were comparable across both methods (Additional file 1: Figure S5). However, scMET outperformed BB MLE when inferring overdispersion parameters *γ*_*j*_, particularly for small numbers of cells (Additional file 1: Figure S6).

To assess whether the shrinkage introduced by scMET improves overdispersion estimates in real data, we performed down-sampling experiments based on a subset of the dataset introduced by [9]. For this analysis, we focused on 424 inhibitory neurons (a more detailed description is provided in the “Methods” section). We compared estimates obtained using the full and down-sampled datasets (Additional file 1: Figure S7). We observed scMET posterior estimates to be more stable than BB MLE as the sample size decreased, suggesting that scMET leads to more robust inference. This is particularly important for rare cell populations or during early development, where large numbers of cells are difficult to obtain. In combination with the simulation study described above, this showcases the benefits of using a Bayesian hierarchical framework to share information across cells and genomic features.

When comparing scMET to existing DNA methylation analysis tools (primarily developed for bulk assays), it is important to emphasize fundamental differences with respect to the functionality provided by scMET. These are summarized in Additional file 1: Table S1 and in the “Methods” section. In particular, most approaches focus on differences in mean methylation. Among others, these are often based on Fisher’s exact test or on BB models (e.g., DSS; [20]). As seen in Additional file 1: Figure S5, both scMET and BB MLE estimates for mean methylation parameters are comparable. As it can be expected, our comparison with respect to DSS also led to similar results (Additional file 1: Figure S8). When comparing with respect to Fisher’s exact test, we did not observe substantial differences in performance either: scMET was better in terms of the F1-measure and type I error control, but led to more conservative results (Additional file 1: Figure S9 and S10 and Additional file 1: Section S2.4). Therefore, we do not aim to claim overall superiority of scMET for differential mean methylation testing. If a user is *only* interested in differential *mean* methylation, existing approaches such as DSS could be used. Instead, the main advantage of scMET is the ability to robustly perform differential variability (DV) testing and HVF selection (benchmarks for our HVF approach are shown in the next section).

In terms of DV testing, our simulations showed that for small effect sizes we would need more than 200 cells to achieve 50 to 80% power, whereas for features with larger effect sizes we would need around 50 cells per group to achieve 80% power (Additional file 1: Figure S11 and Section S2.4). To assess coverage requirements, we grouped features according to the average coverage CpGs across all cells (Additional file 1: Figure S12). For each feature, we computed the posterior standard deviation for the residual overdispersion parameter *ε*_*j*_. Large values (e.g., above 0.5) indicate higher estimation uncertainty and may affect the robustness of downstream analyses. Based on this analysis, we suggest to exclude regions that have less than 3 CpGs covered (on average across all cells) for relatively large sample sizes (>100 cells), and a higher threshold of 5 CpGs for small datasets (<50 cells).

***scMET improves feature selection for unsupervised analysis of single-cell methylomes from the mouse frontal cortex***

**Fig. 2:**
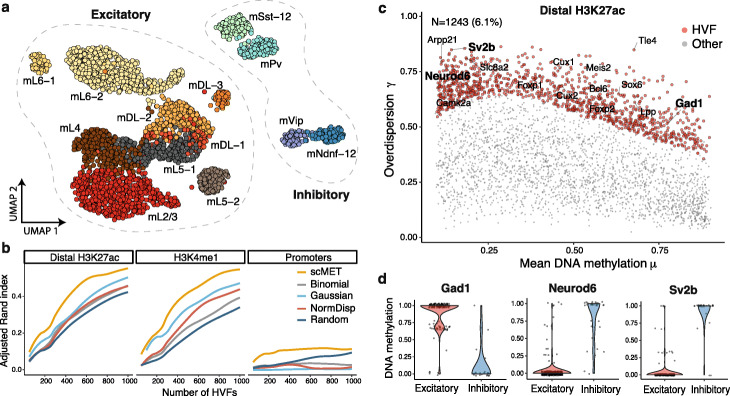
Feature selection using scMET characterises cellular heterogeneity on the mouse frontal cortex. **a** UMAP [26] representation of neuron sub-populations present in the mouse frontal cortex dataset by combining the top 4000 HVFs identified by scMET across distal H3K27ac and H3K4me1 genomic contexts. Cells are colored according to the original cell sub-population assignments obtained by Luo et al. **b** Clustering performance in terms of adjusted Rand index (ARI), for varying number of HVFs. HVF selection was based on scMET’s residual overdispersion parameters *ε*_*j*_ (yellow), binomial variance (gray), Gaussian variance (cyan), normalized dispersion (NormDisp, red), and random selection (blue). A finite grid of HVFs was used for ARI evaluation and non-parametric regression was used to obtain a smoothed interpolation across all values (Methods). **c** Identifying HVFs for the distal H3K27ac genomic context. Red points correspond to features being called as HVF (EFDR = 10% and percentile threshold *δ*_*E*_ = 90%). To ease interpretation, each element is linked to its nearest gene. Labels highlight known neuron marker genes that were used in the [9] study to define the different cell populations. **d** Example distal H3K27ac HVFs whose methylation patterns distinguish the two broad neuronal populations. Panel titles correspond to the nearest genes

To demonstrate the performance of scMET in real data, we considered a dataset where DNA methylation was profiled in 3069 cells isolated from the frontal cortex of young adult mice [9]. To date, this is one of the largest and most heterogeneous publicly available scBS-seq datasets. The main source of heterogeneity in this dataset is due to two broad classes of neurons: excitatory (*I* = 2645) and inhibitory (*I* = 424). Within each class, a hierarchy of sub-populations can be identified according to the cortical depth (Fig. 2a), where excitatory neurons progress from deep layers (mDL-1, mDL-2, mDL-3, mL6-1, mL6-2) to middle (mL5-1, mL5-2, mL4) and superficial layers (mL2/3). These groups were validated in the original study and thus can be used as a benchmark for clustering analyses.

We applied scMET to genomic features from three different putative regulatory elements: gene promoters within 2 kb around transcription start site (*J* = 12,774), distal H3K27ac peaks (*J* = 17,284), and H3K4me1 peaks (*J* = 30,374). As expected, scMET captured the mean-overdispersion relationship within each genomic context, and estimates for residual overdispersion parameters *ε*_*j*_ were not confounded by mean DNAm (Additional file 1: Figure S13).

Here, we illustrate scMET as a feature selection tool, using residual overdispersion estimates to identify HVFs that can be used as input for unsupervised analyses, such as clustering. For each genomic context, we selected HVFs (Additional file 1: Figure S14a) and performed a clustering analysis with varying numbers of HVFs (ranked by decreasing values of their associated tail posterior probabilities) as input. More concretely, we performed dimensionality reduction followed by *k*-means clustering (Methods) and used the adjusted Rand index (ARI, [27]) to quantify agreement with respect to the sub-populations validated by Luo et al. As a comparison, we also evaluated four alternative HVF selection strategies based on a random choice (Random), normalized dispersion values (NormDisp, [28, 29]), Gaussian, and binomial models (Methods). As expected, the clustering performance improved steadily with increasing number of HVFs for all methods. However, scMET consistently led to better clustering performance (Fig. 2b and Additional file 1: Figure S14b, as well as Additional file 1: Figure S15 and S16 for visual inspection in a low-dimensional space). We could already separate inhibitory from excitatory neurons using only the top 100 HVFs obtained by scMET, and generally resulted in more distinct cell sub-populations. In all cases, promoters were less able to disentangle the neuronal sub-populations. This is consistent with the lower overdispersion levels observed in this genomic context (Additional file 1: Figure S14c).

To gain a better understanding about the HVF selection implemented by each method, an additional comparison is provided in Additional file 1: Figure S17a. Similar to scMET, the NormDisp approach selects HVFs that are not confounded by the mean-overdispersion relationship. However, NormDisp HVFs exhibit poorer clustering performance. This potentially occurs due to the normalized dispersion point estimates being more unstable. For instance, when analyzing the discrepancies among the top 200 HVFs selected by NormDisp and scMET, we observe that the top three HVFs called by NormDisp (but not called by scMET) appear to be driven by outliers (Additional file 1: Figure S17b). In contrast, the top HVFs called by scMET (but not called by NormDisp) show a bimodal distribution and are able to better recapitulate the underlying population structure (Additional file 1: Figure S17c).

To facilitate interpretation for the HVFs highlighted by scMET, we linked genomic features to genes by overlapping the genomic coordinates allowing for a maximum distance of 20 kb from the transcription start site in the case of distal elements. We explored whether features identified as HVF (red points in Fig. 2c and Additional file 1: Figure S18a) were enriched for neuronal markers identified in the [9] study (Additional file 2: Table S1). This enrichment was observed for distal H3K27ac and H3K4me1 marks, but not for promoters (Additional file 1: Figure S18b). As representative examples, we display three distal H3K27ac elements among the HVFs that are located proximal to known gene markers of each neuron class: *Gad1* for inhibitory and *Neurod6* and *Sv2b* for excitatory (Fig. 2d).

Finally, we also explored the use of 20-kb sliding windows as input features for scMET (Additional file 1: Section S2.5). After quality control, this led to *J*=82,308 features. HVFs selected among these windows led to similar results as when using pre-annotated H3K27ac and H3K4me1 features as input (Additional file 1: Figure S19a and S19e). This suggests that the selected windows can distinguish most of the different cellular sub-populations. However, the slightly lower ARI values suggest that pre-annotated features can better recapitulate the underlying population structure. Among the windows with the largest residual overdispersion, we identified putative regulatory elements that discriminate cell types and that are located near neuronal marker genes identified by [9] (Additional file 1: Figure S19b-S19d). As an example, chr1:56940001-56960001 is located within the intronic region of *Satb2*, a marker of excitatory neurons. As expected, this region is unmethylated in most excitatory neurons and highly methylated in inhibitory neurons. In contrast, chr1:68780001-68800001 is located within the intronic region of *Erbb4*, a marker of inhibitory neurons. This region is highly methylated in all excitatory neurons and only partially methylated in inhibitory neurons. These results suggests that scMET can provide a meaningful characterization for the role of previously un-annotated genomic regions.

***scMET enables differential methylation testing between cellular sub-populations***

**Fig. 3:**
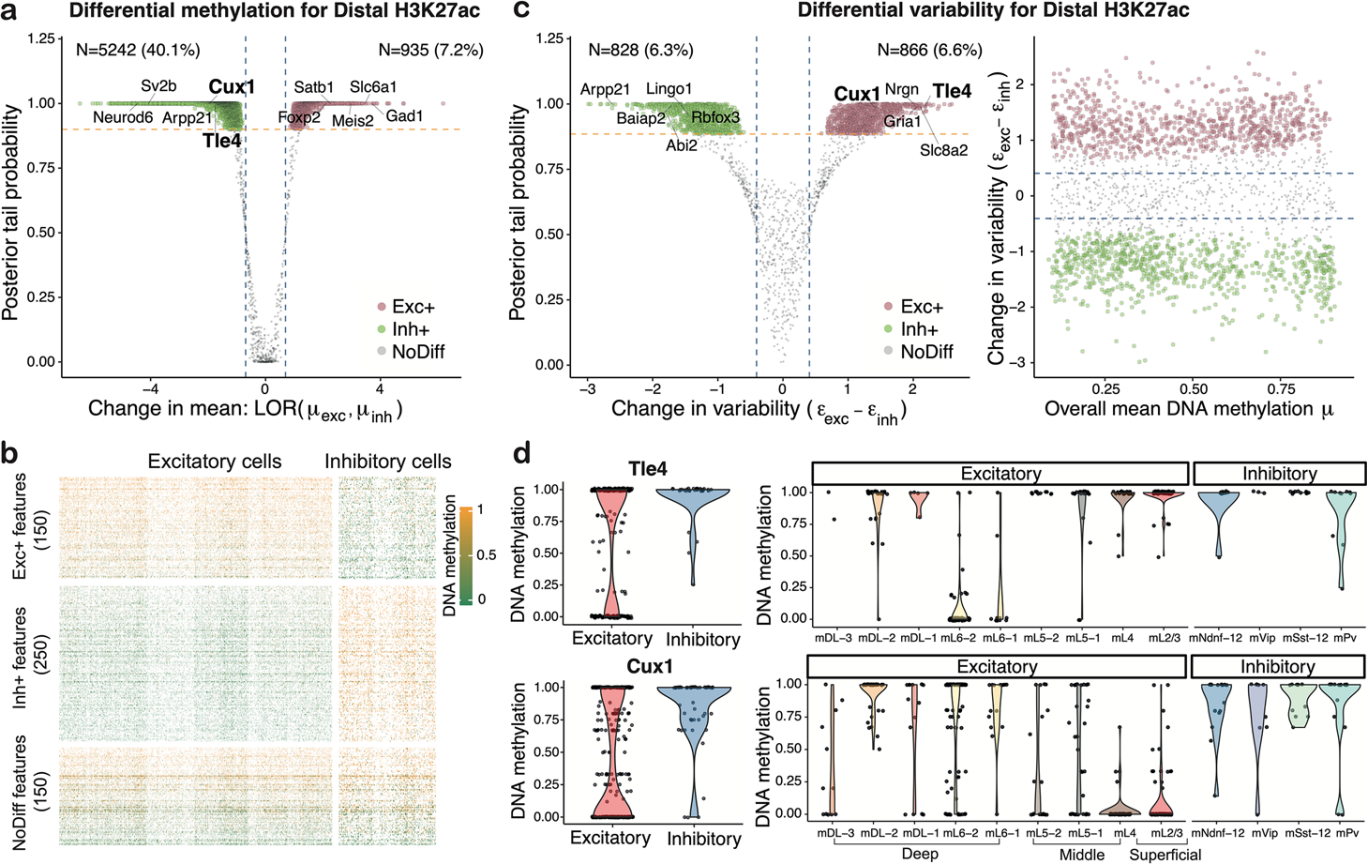
Summary of changes in methylation patterns (mean and variability) for inhibitory and excitatory neurons. **a** Identifying differentially methylated (DM) features for the distal H3K27ac genomic context. Green and pink points correspond to features showing higher mean methylation in inhibitory (Inh+) and excitatory (Exc+) neurons, respectively. Labels highlight neuron marker genes that were used in [9] to define the different cell populations. Blue vertical dashed lines correspond to log-odds ratio threshold *ψ*_*M*_=± log(2). Yellow horizontal dashed line is located at posterior evidence probability cut-offs defined by EFDR = 5%. **b** Representative heatmap of methylation rates (*Y*_*ij*_/*n*_*ij*_) across cells (columns) and features (rows) for the distal H3K27ac genomic context. Cells are grouped in excitatory and inhibitory classes. A set of randomly selected features is displayed. These are grouped according to the DM analysis output as: Exc+, Inh+, and no mean methylation difference. The color code represents features with low (0, green) and high (1, yellow) mean methylation level. Features with no CpG coverage are denoted with white color. **c** Identifying differentially variable (DV) features for the distal H3K27ac genomic context. Green and pink points correspond to features showing higher methylation variability in inhibitory and excitatory neurons, respectively. Blue dashed lines correspond to log-odds ratio threshold *ψ*_*E*_=± log(1.5). Yellow dashed line is located at posterior evidence probability cut-offs defined by EFDR = 5%. For each feature, posterior estimates for the change in residual overdispersion parameter *ε*_*j*_ between excitatory and inhibitory neurons is plotted against the posterior tail probability of calling a feature as DV (left). For each feature, posterior estimates for mean methylation parameter *μ*_*j*_ is plotted against posterior estimates for the change in residual overdispersion parameter *ε*_*j*_ between excitatory and inhibitory neurons (right). **d** Example features that are called as being more variable in excitatory neurons. Left subplots show broad differences in methylation patterns. Right subplots show methylation patterns separately within each broad neuronal class. Each data point represents the methylation rate for a cell

To showcase scMET as a differential methylation tool, we applied it on the same mouse frontal cortex dataset [9], after separating the cells in excitatory and inhibitory groups. Initially, we applied scMET to characterize differential methylation (DM), i.e., changes in mean methylation. Across all genomic contexts, we observed a substantially larger fraction of features being hyper-methylated in inhibitory compared to excitatory neurons (Fig. 3a and Additional file 1: Figure S20a). Within distal H3K27ac peaks, for instance, scMET identified 5242 features to have higher methylation levels in inhibitory neurons, compared to only 935 features showing higher methylation in the excitatory group (Fig. 3a, b). After mapping features to their nearest gene, we observed that DM hits were enriched for known marker genes that differentiate inhibitory and excitatory neurons (Additional file 1: Figure S20b).

Besides DM testing, the primary focus of the scMET differential test is to identify changes in cell-to-cell methylation variability. In principle, differential variability (DV) testing could be based on feature-specific overdispersion parameters *γ*_*j*_, but these results would be confounded by the mean-overdispersion trend (Fig. 1c). Hence, meaningful DV analysis based on *γ*_*j*_ would need to be restricted to non-DM features. Instead, we propose to perform DV analysis based on residual overdispersion parameters *ε*_*j*_. For the mouse frontal cortex dataset, we identified a large number of DV features across genomic contexts, except from promoter regions which showed tighter methylation patterns across inhibitory and excitatory neurons (Fig. 3c and Additional file 1: Figure S21a). Critically, the procedure for calling DV features was not confounded by mean methylation levels (Fig. 3c and Additional file 1: Figure S21b).

As representative examples, we show two distal H3K27ac peaks that are located proximal to neuronal markers and exhibit higher variability in excitatory neurons (Fig. 3d). Both features show substantial variation across the different sub-types of excitatory neurons: the first is mostly unmethylated in the mL6 cortical layer, and the second is mostly unmethylated in the superficial cortical layer. These patterns are consistent with previously reported spatial expression for *Tle4* (mostly expressed in the deep cortical layer, see [30, 31]) and *Cux1* (which shows expression specificity for the superficial layer, see [30, 32]). It should be noted that these features would be excluded from DV analysis based on *γ*_*j*_, since they are also DM between the two broad classes (Fig. 3a). In summary, these findings demonstrate the ability of scMET to identify potential markers that drive between and within cell population heterogeneity.

***Exploring the relationship between transcriptional and DNAm variability using single-cell multi-omics data***

As a second use case, we considered a single-cell multi-omics dataset where scNMT-seq [33] was employed to profile RNA expression, DNAm, and nucleosome occupancy at single-cell resolution, spanning multiple time points from the exit from pluripotency to primary germ layer specification [34]. The multi-modal nature of this dataset provides a unique opportunity to link cell-to-cell variation between DNAm and transcription across individual cells. Here, we used scMET to quantify DNAm variability at promoter elements, which we subsequently contrasted to RNA expression heterogeneity for the corresponding genes. For this analysis, we exclusively used promoter elements as, unlike distal regulatory elements, they can be unambiguously matched to their respective genes.

For each gene, we quantified transcriptional heterogeneity using the residual overdispersion estimates generated by BASiCS (Additional file 1: Figure S22a, [22]). Promoter DNAm variability was calculated using the residual overdispersion estimates inferred using scMET (Additional file 1: Figure S22b). More details about these analyses and the associated data pre-processing steps are described in the “Methods” section.

**Fig. 4:**
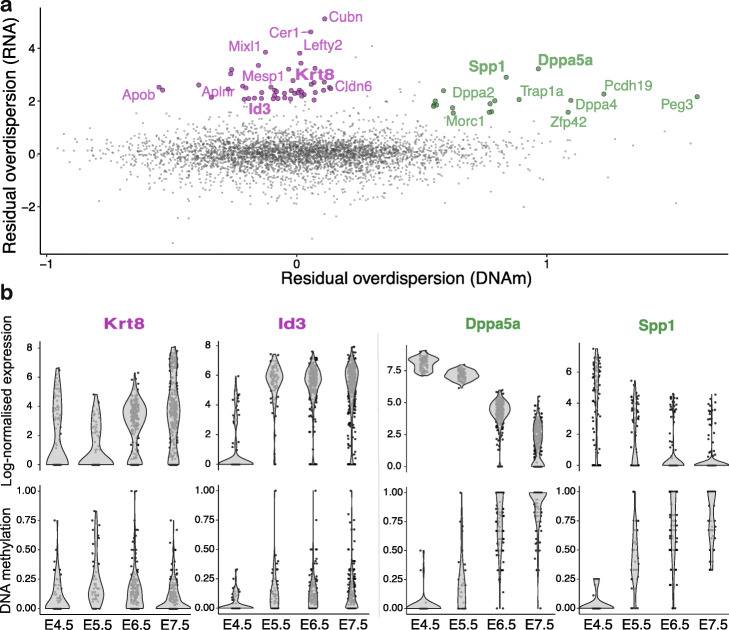
scMET applied to the multi-omics scNMT-seq gastrulation dataset reveals a complex linkage between promoter DNAm and RNA expression during embryonic development. **a** Scatter plot displays posterior median estimates for residual DNAm overdispersion parameters *ε*_*j*_ in gene promoters (*x*-axis) versus RNA residual overdispersion of the corresponding genes (*y*-axis). Among the genes with high levels of RNA heterogeneity, green and pink points correspond to promoters showing high and low levels of DNAm variability, respectively. **b** Representative examples of DNAm and RNA expression patterns across developmental stages for genes with high transcriptional heterogeneity and low (left, pink) or high (right, green) DNAm heterogeneity. The *y*-axis shows BASiCS log-normalized gene expression (in a log(*x*+1) scale) (top) and promoter DNAm rate (bottom). Cells are stratified by embryonic stage (*x*-axis)

When comparing residual overdispersion estimates for RNA expression and promoter DNAm, there was no clear genome-wide association (Fig. 4a). However, when restricting to genes that display high levels of transcriptional variability, two main groups can be identified. The first category corresponds to genes with low levels of promoter DNAm residual overdispersion, and it includes differentiation and germ layer markers such as *Mesp1*, *Lefty2*, and *Id3* (mesoderm) and *Cldn6*, *Cer1*, and *Krt8* (endoderm). The second category is characterized by genes with high promoter DNAm residual overdispersion and includes known pluripotency markers such as *Dppa5a*, *Zfp42*, *Spp1*, and *Peg3*. Representative examples for these genes are displayed in Fig. 4b.

These results suggest the presence of two modes of regulation. On the one hand, downregulation of pluripotency genes is associated with high promoter DNAm heterogeneity, linked to a pronounced increase in promoter DNA methylation throughout the embryonic stages. On the other hand, upregulation of differentiation genes is not linked to high levels of promoter DNAm variability. This suggests that other genomic contexts or molecular layers might be responsible for their activation [34]. Finally, we also find genes with low RNA expression variability that display high levels of promoter DNAm heterogeneity (Additional file 1: Figure S22c), suggesting that the coupling between promoter DNAm and transcriptional activity is more complex than previously acknowledged during embryonic development stages [35].

## Discussion

Single-cell DNAm assays can currently profile hundreds to thousands of DNA methylomes, with increasingly complex experimental designs. The high resolution of these measurements enables us to measure cell-to-cell epigenetic variability, as well as uncover the regulatory features that modulate it [36]. However, the noise and biases intrinsic to such technologies create a need for computational frameworks that can systematically interrogate the data generated, dissecting genuine variability and quantifying uncertainties.

In this study, we introduced scMET, a statistical framework for modeling DNA methylation heterogeneity from scBS-seq data. Using a hierarchical Bayesian framework to borrow information across cells and features, scMET robustly quantifies genuine cell-to-cell variability. Our results demonstrated the ability of scMET in highlighting genomic features that drive cell-to-cell heterogeneity across neuronal sub-populations in a large dataset of single-cell methylomes from the mouse frontal cortex. Furthermore, scMET can be used as a quantitative tool to interrogate changes in DNAm patterns between pre-specified cell populations. Unlike common approaches that only detect changes in mean methylation levels [20, 37], scMET can also identify features with differences in DNAm variability between populations. Importantly, the differential variability estimates are quantified through residual overdispersion parameters, thus accounting for the known confounding relationship between mean and overdispersion in scBS-seq datasets.

Recently, complementary approaches have been proposed to analyze single-cell DNAm data. These include Melissa [13] and Epiclomal [38], which are probabilistic clustering and imputation methods based on a hierarchical mixture model. In addition, MAPLE [39] uses a supervised learning approach to detect correlations between RNA expression and DNAm, which enables cell type assignments by transferring labels from scRNA-seq to single-cell DNAm datasets. In all cases, scMET could be incorporated in the analysis pipeline, by selecting a relevant set of input features defined by identifying regions with high DNAm variability which can better characterize the underlying population structure.

scMET uses a GLM framework to explicitly model known biases in the data in the form of additional covariates, such as CpG content. The flexibility of the GLM approach enables it to easily incorporate additional features, such as DNA motifs, which could be important to elucidate the role of sequence or chromatin state in modulating DNA methylation. Additionally, the framework could readily be extended to model joint variability in multiple molecular layers (such as transcriptome and methylome), opening a path to new methodologies in integrative, single-cell multi-omics analyses. Given the increasing prominence of such studies, we expect scMET to become an important tool in the extraction of biological signals from DNAm datasets of increasing complexity.

## Conclusions

We presented scMET — a Bayesian hierarchical approach to robustly quantify epigenetic heterogeneity using high-throughput single-cell DNAm datasets. We extensively evaluated the performance of scMET using synthetic data and recent large-scale assays in the context of the mouse brain cortex and during early development. In particular, we have shown how feature selection by scMET can be incorporated in single-cell DNAm analysis pipelines, improving the characterization of epigenetically distinct cell types. Moreover, we introduced a novel differential methylation framework which, unlike existing approaches, fully exploits the cellular resolution of the data to identify changes in epigenetic variability. In combination, these findings demonstrate how the integrative approach implemented in scMET can overcome data sparsity and improve the quantification of genuine epigenetic cell-to-cell heterogeneity.

## Methods

### The scMET model

Let *Y*_*ij*_ represent the number of methylated CpGs out of the *n*_*ij*_ CpGs for which DNAm was measured for genomic feature *j*∈{1,…,*J*} in cell *i*∈{1,…,*I*} (see Additional file 1: Figure S1). These genomic features could be defined by pre-annotated regions (e.g., enhancers) or other regions of interest. To capture data overdispersion, scMET assumes a beta-binomial (BB) hierarchical formulation (see also Additional file 1: Section S2.1):

1 $$ \begin{aligned} Y_{ij} \ |\ \theta_{ij} \sim \text{Binomial} (n_{ij}, \theta_{ij}), \qquad \theta_{ij} \ |\ \mu_{j}, \gamma_{j} \sim \text{Beta} (\mu_{j}, \gamma_{j}). \end{aligned}  $$

In Eq. (1), the beta distribution is parameterized such that $\mathbb {E}[\theta _{ij}] = \mu _{j}$ and $\text {Var}[\theta _{ij}] = \gamma _{j}^{2}\mu _{j}\left (1 - \mu _{j}(1 - \gamma _{j}) - \gamma _{j}\right) (1 - \gamma _{j})^{-1}$, with *μ*_*j*_∈(0,1) and *γ*_*j*_∈(0,1). If *γ*_*j*_=0, the model in Eq. (1) reduces to a binomial model with parameters *n*_*ij*_ and *μ*_*j*_. After integrating out the random effects *θ*_*ij*_, it can be seen that *μ*_*j*_ corresponds to the mean methylation across all cells for feature *j* and that *γ*_*j*_ controls the *overdispersion* that is not captured by binomial sampling. In fact, the BB variance can be written as:

2 $$ \text{Var}[Y_{ij} \ |\ \mu_{j}, \gamma_{j}] = \underbrace{n_{ij}\mu_{j}(1 - \mu_{j})}_{\text{technical variation}} + \underbrace{n_{ij}\mu_{j}(1 - \mu_{j}) (n_{ij} - 1)\gamma_{j}}_{\text{additional (biological) variation}}.  $$

Parameters *μ*_*j*_ and *γ*_*j*_ can be inferred via maximum likelihood estimation. However, due to the high sparsity and noise present in single-cell DNAm data, these estimates can be unstable, especially for overdispersion parameters *γ*_*j*_ (Additional file 1: Figure S6 and S7). To overcome this, we use a Bayesian framework with a hierarchical prior specification for *μ*_*j*_ and *γ*_*j*_, sharing information across sets of similar types of genomic features (e.g., enhancers). Our approach is flexible and can incorporate feature-specific covariates **x**_*j*_ that explain differences in mean methylation across features. For instance, features with high CpG density tend to have lower methylation levels. These covariates are introduced within a generalized linear model (GLM) framework through the prior on the mean methylation parameters *μ*_*j*_:

3 $$  \mu_{j} \ |\ \mathbf{w}_{\mu}, s_{\mu}, \mathbf{x}_{j} \sim \text{Logit}\mathcal{N} \left(f_{\mu}(\mathbf{x}_{j}; \mathbf{w}_{\mu}), s_{\mu}\right), \quad \text{where}\; f_{\mu}(\mathbf{x}_{j}; \mathbf{w}_{\mu}) = \mathbf{w}_{\mu}^{\top}\mathbf{x}_{j}.  $$

In Eq. (3), $\text {Logit}\mathcal {N}$ denotes a logit-normal distribution, **w**_*μ*_ is a vector of regression coefficients, and *s*_*μ*_ is the standard deviation for logit(*μ*_*j*_). Throughout our analyses, we assume **x**_*j*_=(1,*C*_*j*_), where *C*_*j*_ denotes the CpG density for feature *j*. However, scMET is flexible and users can introduce other feature-specific covariates.

Our prior specification is also designed to capture the mean-overdispersion relationship that is typically observed in the data generated by high-throughput sequencing assays, such as scBS-seq (Fig. 1c). Here, we follow the approach in [22], introducing a non-linear regression model through an informative prior for *γ*_*j*_:

4 $$ \gamma_{j} \ |\ \mu_{j}, \mathbf{w}_{\gamma}, s_{\gamma} \sim \text{Logit}\mathcal{N} (f_{\gamma}(\mu_{j}; \mathbf{w}_{\gamma}), s_{\gamma}), \quad \text{where}\; f_{\gamma}(\mu_{j}; \mathbf{w}_{\gamma}) = w_{\gamma 1} + \sum_{l=2}^{L} w_{\gamma l} g_{l}(\mu_{j}).  $$

Here, *f*_*γ*_(*μ*_*j*_;**w**) can be interpreted as the overdispersion (logit scale) that is predicted by mean methylation levels *μ*_*j*_ (fitted black line in Fig. 1c), *g*_*l*_(*μ*_*j*_) represent radial basis function kernels (defined as in [40]), and *w*_*γ*1_,…,*w*_*γ*,*L*_ are regression coefficients. Unless otherwise stated, we use *L*=4 throughout our analyses. The remaining elements of the prior are described in Additional file 1: Section 2.2.

The prior distribution in Eq. (4) can be rewritten as a non-linear regression model

5 $$  \text{logit}(\gamma_{j}) = f_{\gamma}(\mu_{j}; \mathbf{w}_{\gamma}) + \epsilon_{j}, \quad \epsilon_{j} \sim \mathcal{N}(0, s_{\gamma}),  $$

where *ε*_*j*_ corresponds to a feature-specific *residual overdispersion* parameter that captures deviations from the overall trend. Hence, a feature that exhibits positive *ε*_*j*_ values has more variation than expected for features with similar mean methylation. Accordingly, negative *ε*_*j*_ values indicate less variation than expected for features with similar mean methylation.

#### Implementation

The posterior distribution for the model parameters in scMET is not amenable to analytic solutions. Hence, we resort to variational Bayes (VB, [23]) and Markov Chain Monte Carlo (MCMC, [41]) implementations using the Stan probabilistic programming language [42]. scMET is publicly available as an R package at https://github.com/andreaskapou/scMET and will be shortly submitted to Bioconductor.

#### Identifying highly variable features

Residual overdispersion parameters *ε*_*j*_ can be used to label highly variable features (HVFs) within a population of cells. Our decision rule is based on tail posterior probabilities [24] associated to whether *ε*_*j*_ exceed a pre-specified threshold *ε*_0_:

6 $$ \pi_{j}^{E}\left(\epsilon_{0}\right) \equiv P(\epsilon_{j} > \epsilon_{0} \ |\ \text{data}),  $$

As a default choice, we define *ε*_0_ based on the distribution of posterior estimates for residual overdispersion parameters *ε*_*j*_ across all features. In particular, we define *ε*_0_ to match the *δ*_*E*_-th percentile of the distribution. Unless otherwise stated, we set as default *δ*_*E*_=0.9.

The probabilities in Eq. (6) can be estimated by counting the proportion of posterior draws (obtained by VB or MCMC) for which the chosen criteria are met [43]. scMET labels as HVFs those for which their associated tail posterior probabilities are above a given posterior evidence threshold *α*_*H*_(0.6<*α*_*H*_<1), where *α*_*H*_ is calibrated via the expected false discovery rate (EFDR; [25]), see also Additional file 1: Section S2.3.

#### Differential testing

scMET provides a similar probabilistic rule to label differentially methylated (DM) and differentially variable (DV) features across experimental conditions or cell types (Fig 1e). Here, we define DM features as those for which mean methylation varies across the groups of cells under study. More concretely, let $\mu ^{A}_{j}$ and $\mu ^{B}_{j}$ be the mean methylation parameters associated with feature *j* in groups A and B. We quantify differences in mean methylation as the log-odds ratio (LOR):

7 $$ \text{LOR}(\mu^{A}_{j}, \mu^{B}_{j}) = \text{logit}(\mu^{A}_{j}) - \text{logit}(\mu^{B}_{j}).  $$

Similar to the HVF analysis, our decision rule for DM testing is defined as:

8 $$ \pi_{jAB}^{M}\left(\psi_{M}\right) \equiv P(|\text{LOR}(\mu_{j}^{A}, \mu_{j}^{B})| > \psi_{M} \;|\; \text{data}) > \alpha_{M},  $$

where *α*_*M*_(0.6<*α*_*M*_<1) is a posterior evidence threshold chosen to match a desired EFDR level and *ψ*_*M*_ is a LOR threshold which can be interpreted as a minimum effect size to be detected by the test. As default, we use *ψ*_*M*_= log(2), i.e., a two-fold change in odds ratio.

Beyond highlighting DM features, scMET embraces the cellular resolution of scBS-seq data to perform differential variability (DV) analyses, identifying changes in cell-to-cell DNAm variability across groups. Although overdispersion parameters *γ*_*j*_ could be used as the input for the DV test, the results would be confounded by the mean-overdispersion relationship that is typically observed within each genomic context (Fig. 1c). Instead, we propose to perform DV analysis based on *ε*_*j*_ — a measure of cell-to-cell DNAm variability that is not confounded by differences in mean methylation. Let $\gamma _{j}^{A}$ and $\gamma _{j}^{B}$ denote the overdispersion parameters linked to feature *j* in groups A and B. To label DV features based on residual overdispersion, we make use of Eq. (5), and decompose the LOR between $\gamma _{j}^{A}$ and $\gamma _{j}^{B}$ parameters as:

9 $$  \text{LOR}(\gamma_{j}^{A}, \gamma_{j}^{B}) = \underbrace{f_{\mu}^{A}(\mathbf{x}_{j}; \mathbf{w}_{\mu}^{A}) - f_{\mu}^{B}(\mathbf{x}_{j}; \mathbf{w}_{\mu}^{B})}_{\text{mean contribution}} + \underbrace{\epsilon_{j}^{A} - \epsilon_{j}^{B} }_{\text{residual change}}.  $$

In Eq. (9), the first term captures the changes in overdispersion that are explained by mean methylation and the second term captures residual overdispersion changes after accounting for the mean methylation. This residual change is used to identify features with statistically significant differences in residual overdispersion. For a given posterior evidence threshold *α*_*E*_(0.6<*α*_*E*_<1) and tolerance threshold *ψ*_*E*_, the following rule is used to identify DV features:

10 $$ \pi_{jAB}^{E}\left(\psi_{E}\right) \equiv P(|\epsilon_{j}^{A} - \epsilon_{j}^{B}| > \psi_{E} \;|\; \text{data}) > \alpha_{E}.  $$

As default, we set *ψ*_*E*_= log(1.5), i.e., 50% change in overdispersion LOR between the groups. As above, the posterior evidence threshold *α*_*E*_ is calibrated via the EFDR, see Additional file 1: Section 2.3.

### Alternative methods

When comparing scMET to existing DNAm analysis tools (primarily developed for bulk assays), it is important to emphasize fundamental differences with respect to the functionality provided by scMET. These are summarised in Table S1 and briefly described below.

#### Parameter estimation

The BB MLE method corresponds to estimating the parameters of the beta-binomial model in Eq. (1) independently per feature using maximum likelihood. The VGAM package was used for parameter estimation [44].

#### Differential mean methylation analyses

When comparing methylation profiles between experimental conditions or cell types, bulk DNA methylation analysis tools primarily focus on differences in mean methylation. Existing methods are often based on some version of a *t*-test (e.g., DMRcate [45], BSmooth [37]); a Fisher test on the methylation counts (e.g., MethylKit [46], RnBeads [47]); negative binomial models (often adapted from the bulk RNA sequencing literature, e.g., edgeR [48]); or on beta-binomial models (e.g., DSS [20], RADMeth [21]).

For differential mean methylation testing on the synthetic datasets, we compared scMET with Fisher’s exact test. Features with log-odds ratio > log(1.5) between the two groups and FDR <10% (Benjamini-Hochberg procedure) were called as differentially methylated.

#### Differential methylation variability analyses

In the context of microarray DNAm, DiffVar [49] was proposed to perform DNAm variability analyses, with an empirical Bayes framework to stabilize the t-statistics when contrasting the variance of the continuous methylation rates. This approach can also be applied to bulk bisulphite data, as methylation rates can be confidently estimated when having a large number of observations. However, this is not appropriate for sparse measurements (i.e., in single-cell data), as there is high uncertainty in their estimation. Instead, scMET directly models methylation read-counts, using a hierarchical framework to address data sparsity and to propagate statistical uncertainty. It is also important to note that single-cell bisulphite data displays a strong dependency between mean and variability estimates (see, e.g., Additional file 1: Figure S13 and S23). Hence, direct downstream analyses of methylation variability would be confounded by mean methylation. Instead, scMET explicitly derives a residual measure of variability (residual overdispersion) that is not confounded by mean methylation and that can be directly employed in downstream tasks such as HVF selection and differential variability.

#### HVF selection

Feature selection has traditionally been done either using the binomial variance or the Gaussian variance as variability estimates [33, 50]. The binomial model is where features are ranked according to binomial variance given by $1/I\sum _{i}\theta _{ij} (1 - \theta _{ij})$, where *θ*_*ij*_=*Y*_*ij*_/*n*_*ij*_ is the methylation rate for feature *j* in cell *i*. The Gaussian model on methylation rates $\theta _{ij} \sim \mathcal {N}(\mu _{j}, \sigma _{j})$ is where features are ranked according to *σ*_*j*_. The normalized dispersion (NormDisp) method is widely applied for scRNA-seq data [28, 29]. Briefly, we calculated the mean and a dispersion measure (variance/mean) for each gene across all single cells and placed genes into 20 bins based on their mean expression. Normalized dispersion is calculated as the absolute difference between dispersion and median dispersion of the expression mean, normalized by median absolute deviation within each bin [29]. Additionally, we also include a random selection approach in which HVFs are selected at random among all input features. Finally, we note that, to our knowledge, none of the beta-binomial or negative binomial methods discussed above implements a strategy to perform HVF selection.

### Scalable strategy for genome-wide sliding windows

By pooling information across cells and features, scMET leads to more robust parameter estimates than simpler methods which analyze each feature independently. We addressed the increased computational complexity by adopting a variational Bayes algorithm which scales linearly with the number of features, enabling the analysis of large-scale data sets (Additional file 1: Figure S2). However, additional challenges arise due to the increased dimensionality introduced by using a sliding window approach on a genome-wide scale. For example, for the mouse genome, using sliding windows of 20 kb with a step size of 20 kb yields approximately 130,000 features. This may preclude the use of scMET within a practical timeline for large-scale applications. In the spirit of divide-and-conquer schemes [51], we bypass this problem via a parallelization strategy in which we apply scMET separately to each chromosome. Feature-specific estimates obtained for each chromosome can be combined post hoc when performing HVF selection and differential analyses.

We suggest careful consideration when selecting the input feature set while using the sliding window approach. First, it is critical to apply a quality control step to remove windows with very low coverage (number of CpGs whose methylation status was observed), for which inference is less reliable. Second, the choice of window size can restrict the type of regions to be included in the analysis. For example, regulatory elements such as enhancers tend to be relatively small, spanning from a hundred to a few thousand base pairs [52]. Such regions would be excluded when using large window sizes. In contrast, small windows may fail to have sufficient coverage. Data-driven approaches could be used to identify an optimal window size. For example, [53] identified that 2-kb windows are sufficient to capture regions with high CpG-to-CpG concordance. An alternative solution would be to iteratively refine the window size while performing downstream analyses. This was implemented in *metilene* [54] in the context of differential mean methylation testing for bulk DNAm sequencing data. Implementation of such iterative strategy for differential variability testing and HVF selection while taking into account the sparsity of single-cell DNAm data is a very interesting direction for future research.

### Simulation study

We simulated *J* = 300 features for varying number of cells ranging from *I* = 20 up to *I* = 1000. To mimic the properties observed in real scBS-seq data, we assume that for each feature we have coverage for a subset of cells given by *I*_*j*_∼Binomial(*I*,*p*_*j*_), where *p*_*j*_∼Uniform(0.4,0.8) to generate diverse *I*_*j*_ across features. We also simulate three alternative regions that have rich (*N* = 50), moderate (*N* = 15), and poor (*N* = 8) CpG density. That is, the number of CpGs (*n*_*ij*_) is simulated from Binomial(*N*,*q*_*j*_), where *q*_*j*_∼Uniform(0.4,0.8) to generate a broad range of CpG coverage across features. Next, for each feature, we generate mean methylation parameters $\mu _{j} \sim \text {Logit}\mathcal {N}(\mathbf {w}_{\mu }^{\top }\mathbf {x}_{j}, 1)$, where **w**_*μ*_=(−0.5,−1.5) and **x**_*j*_=(1,*C*_*j*_) are feature-specific covariates, where *C*_*j*_ denotes the CpG density. The negative weight on **w**_*μ*_ is used to simulate the known negative association between mean methylation and CpG density. Next, we simulated feature-specific overdispersion parameters $\gamma _{j} \sim \text {Logit}\mathcal {N}(\mathbf {w}_{\gamma }^{\top }\mathbf {g}_{j}(\mu _{j}), 0.25)$ to mimic the mean-overdispersion relationship. We set **w**_*γ*_=(−1.2,−.3,1.1,−.9) and **g**_*j*_(*μ*_*j*_) is a vector of basis functions with methylation level *μ*_*j*_. Finally, we simulated the number of methylated CpGs from BB distribution, using the VGAM package, that is, *Y*_*ij*_∼BB(*n*_*ij*_,*μ*_*j*_,*γ*_*j*_).

For differential testing analysis, we used the above approach to generate cells from the first group (group A). For DM analysis, 15% of features were randomly selected and their corresponding *μ*_*j*_ were randomly increased or decreased by three different LOR thresholds: 2, 3, and 5, to generate cells from the second group (group B). Similarly, for DV analysis, 15% of features were randomly selected from the first group and their corresponding *γ*_*j*_ were randomly increased or decreased by three different LOR thresholds: 2, 3, and 5, to generate cells from the second group.

### Mouse frontal cortex dataset

#### Data processing

The dataset is available from the Gene Expression Omnibus repository under accession number GSE97179. Details on quality control and data pre-processing can be found in [9]. Additional file 3: Table S2 contains metadata for the 3069 cells, such as cell type annotations and cortical layer information. We aggregated closely related cellular sub-populations with less than 25 cells, following the hierarchy established in [9]. DNA methylation was quantified using mCG dinucleotides over three genomic contexts: (1) gene promoters (±2-kb windows around the transcription start sites of genes extracted from ENSEMBL version 87, [55]), (2) Distal H3K27ac ChIP-seq peaks, and (3) H3K4me1 ChIP-seq peaks. The latter was based on two ChIP-seq datasets that were profiled in adult (8 weeks) mouse cortex as part of the ENCODE project (see Additional file 4: Table S3).

For each genomic feature *j*∈{1,…,*J*} and cell *i*∈{1,…,*I*}, the following censoring procedure was applied: we recorded *Y*_*ij*_ as a missing value if methylation coverage was available in less than 3 CpGs (i.e., *n*_*ij*_<3). The purpose of this censoring step was to exclude observations with very low coverage for which DNAm quantification is less reliable. Subsequently, we removed features that did not have CpG coverage in at least 15 cells. In addition, we excluded features that had mean methylation across cells lower than 0.1 or higher than 0.9; the rationale being that fully (un)methylated features do not drive methylation heterogeneity and will not provide information for identifying cell sub-populations.

#### Down-sampling experiment

Using the characterized sub-populations from [9], we performed down-sampling experiments on 424 inhibitory neurons. Both scMET and BB MLE methods were run once on the full dataset (424 cells) to generate pseudo-ground truth parameter estimates. Subsequently, 20, 50, 100, and 200 cells were randomly down-sampled from the full population prior to parameter estimation. This procedure was repeated 5 times for each sample size. The same censoring step as described above was applied. Moreover, due to smaller sample sizes, we filtered genomic features that did not have CpG coverage in at least 5 cells.

#### HVF analysis

HVF analysis was applied on 12,774 gene promoters, 17,284 distal H3K27ac peaks, and 30,374 H3K4me1 peaks. To model the mean-overdispersion relationship, we used *L* = 4 radial basis function kernels and kept default hyper-parameter values (Additional file 1: Section S2.2). The total number of iterations was set to 50,000 and convergence was attained when the evidence lower bound difference between two consecutive iterations was less than 1e −04.

#### Differential analysis

For differential analysis between excitatory and inhibitory neurons, we only included features with CpG coverage in at least 15 cells, in both sub-populations. This resulted in 12,611 gene promoters, 13,075 distal H3K27ac peaks, and 20,212 H3K4me1 peaks. To model the mean-overdispersion relationship, we used *L* = 4 radial basis function kernels and kept default hyper-parameter values (Additional file 1: Section S2.2). The total number of iterations was set to 50,000 and convergence was attained when the evidence lower bound difference between two consecutive iterations was less than 1×10^−4^.

#### Dimensionality reduction

Dimensionality reduction was applied using a Bayesian Factor Analysis algorithm, as implemented in the MOFA2 package [56]. The motivation for this method, as opposed to the conventional Principal Component Analysis, is to handle the large presence of missing values without the need for imputation. A second (non-linear) dimensionality reduction step was applied using UMAP (as implemented in the umap package) to project the data into a two-dimensional space (Additional file 1: Figure S15 and S16).

#### Clustering

A finite grid of HVFs (from 50 to 1000 with step size of 50) was selected by each of the competing methods. Subsequently, clustering analysis was performed using the *k*-means algorithm on the latent space defined by the MOFA factors (fixed to 15). The number of clusters was set to the number of cell types as characterized by [9]. We assessed clustering performance using the ARI and cluster purity. A non-parametric regression (implemented by the loess function) was used to obtain a smoothed interpolation across all HVF values.

### scNMT-seq gastrulation dataset

#### Data processing

The parsed scNMT-seq gastrulation dataset was downloaded from https://ftp://ftp.ebi.ac.uk/pub/databases/scnmt_gastrulation. Raw files are available from the Gene Expression Omnibus repository under accession number GSE121708. Details on the quality control and data processing can be found in [34]. We selected all cells from E4.5 to E7.5 days after excluding the extra-embryonic visceral endoderm cells, as they display distinct DNA methylation profiles. Additional file 5: Table S4 contains sample metadata for the 848 cells retained for analysis. DNA methylation was quantified over gene promoters (±2kb windows around the transcription start sites of genes extracted from ENSEMBL version 87, [55]).

#### Calculation of DNA methylation and RNA expression heterogeneity

For the DNAm data, we applied the same censoring procedure and feature exclusion criteria as described in the pre-processing of the [9] dataset. This resulted in 13,785 gene promoters for downstream analysis. Residual overdispersion estimates were calculated by scMET with default parameter values using the same number of iterations and convergence criteria described above.

For the RNA expression data, we removed lowly expressed genes (no counts in less than 10 cells and average count across expressed cells less than 5). This resulted in 14,076 genes for downstream analysis. Residual overdispersion estimates were obtained using BASiCS [22]. The algorithm was run using 20,000 iterations, applying a burn in of 10,000 and thinning of 10. An empirical Bayes approach was used to derive the prior hyper-parameters associated to gene-specific mean expression parameters within BASiCS. In the comparison displayed in Fig. 4, we focused on the 10,192 genes contained in the intersection of the lists obtained above.

## Supplementary Information

**Additional file 1** Supplementary material. Supplementary figures (Section 1) and supplementary notes (Section 2).

**Additional file 2** **Table S1**. Table with marker genes from the Luo2017 [9] study.

**Additional file 3** **Table S2**. Table with sample metadata from the Luo2017 [9] study.

**Additional file 4** **Table S3**. Table with ChIP-seq annotation information.

**Additional file 5** **Table S4**. Table with sample metadata from the Argelaguet2019 [34] study.

**Additional file 6** Review history.

## Data Availability

All datasets analyzed in this article are publicly available. The mouse cortex dataset [9] is available under GEO accession number GSE97179. The parsed scNMT-seq gastrulation dataset [34] was downloaded from https://ftp://ftp.ebi.ac.uk/pub/databases/scnmt_gastrulation. Raw files are available under GEO accession number GSE121708. The scMET model is publicly available as R software released under the GNU GPL-3 license (see https://github.com/andreaskapou/scMET [57] and 10.5281/zenodo.4629327 [57]). The code used to process and analyze the data is available at https://github.com/andreaskapou/scMET-analysis. The following software versions were used throughout the analyses: scMET (0.99.1), BASiCS (2.0.1), coda (0.19.3), MOFA2 (0.99.7), rstan (2.19.3), uwot (0.1.10), and VGAM (1.1.3). Declarations
