# Supplementary material for: scMET: Bayesian modeling of DNA methylation heterogeneity at single-cell resolution
Source: Genome Biol. 2021 Apr 20;22:114. doi: 10.1186/s13059-021-02329-8 (PMC8056718; doi:10.1186/s13059-021-02329-8)
Supplement: Supplementary file 1 — Additional file 1 Supplementary material. Supplementary figures (Section 1) and supplementary notes (Section 2). [file 13059_2021_2329_MOESM1_ESM.pdf]

Supplementary material for

scMET: Bayesian modelling of DNA methylation heterogeneity at single-cell resolution

Chantriolnt-Andreas Kapourani[1,2,*] Ricard Argelaguet[3,*] Guido Sanguinetti[2,4,†]
Catalina A. Vallejos[1,5,†]

[1]MRC Institute of Genetics and Molecular Medicine, University of Edinburgh, UK
[2]School of Informatics, University of Edinburgh, UK
[3]European Bioinformatics Institute (EMBL-EBI), Hinxton, UK
[4] SISSA, International School of Advanced Studies, Trieste, Italy
[5] The Alan Turing Institute, London, UK
[*]These authors contributed equally [†]Corresponding author
Email: catalina.vallejos@igmm.ed.ac.uk (C.A.V.); gsanguin@sissa.it (G.S.)

S1 Supplementary figures

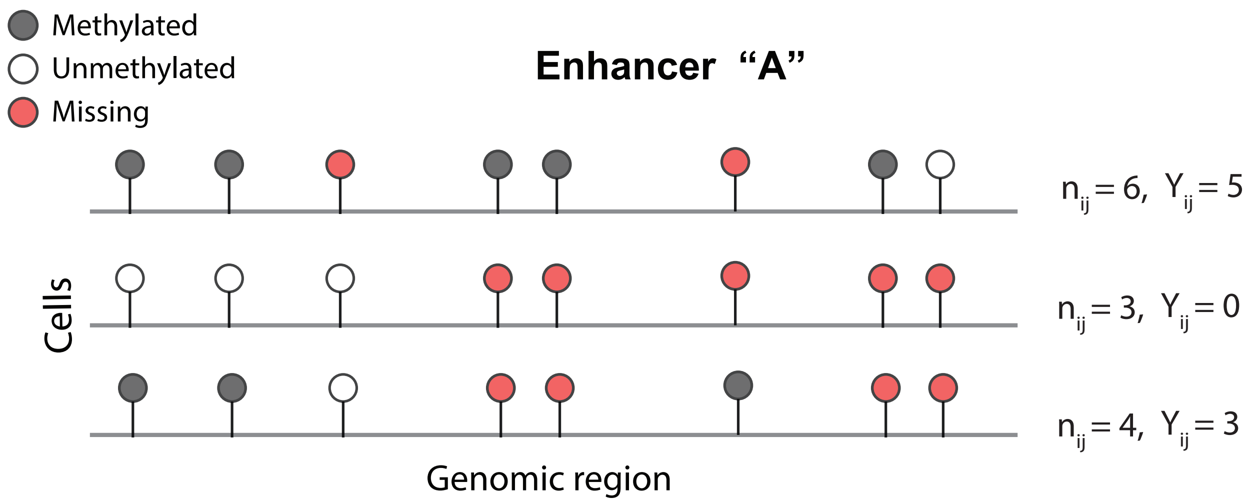

Figure S1: For each region of interest (e.g. enhancer 'A'), the input data corresponds to the number of CpG sites for which a valid measurement was recorded (n_{ij}) and, among those, the number of methylated CpG sites Y_{ij}. Note that many CpG sites will not becovered by a read (denoted by red colour), leading to sparse information per genomic region. Each horizontal line denotes a genomic region across different single cells.

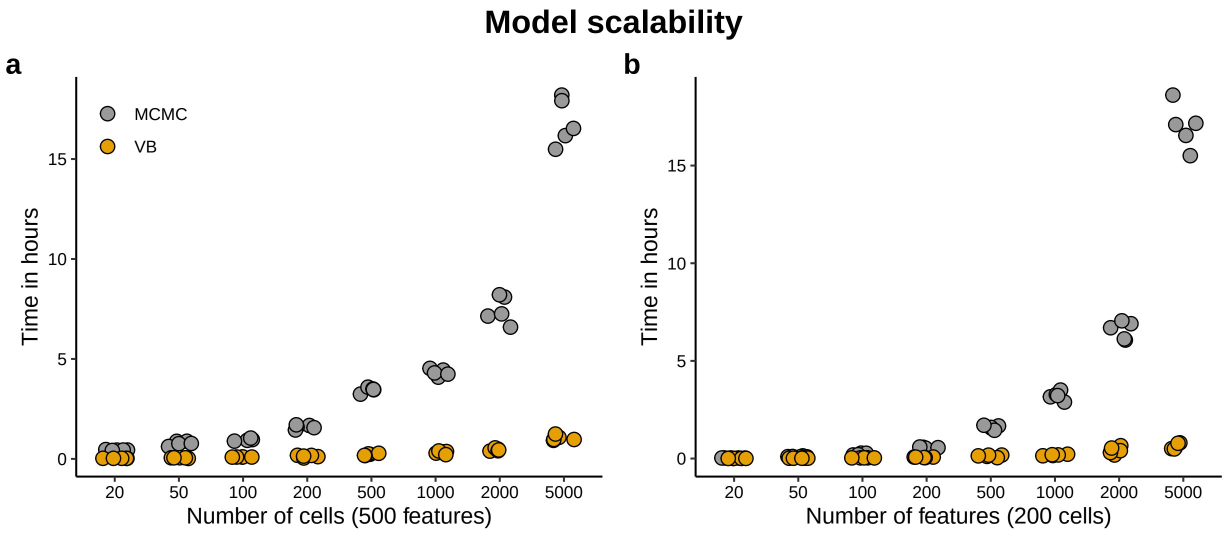

Figure S2: scMET scalability analysis using synthetic data. Running times for varying number of (**a**) cells and (**b**) features. Each dot represents a different run comparing variational Bayes (VB, yellow) and Markov Chain Monte Carlo (MCMC, grey) implementations of scMET in Stan (1). Total number of iterations for VB was set to a maximum of 20,000, whereas for MCMC to 3,000.

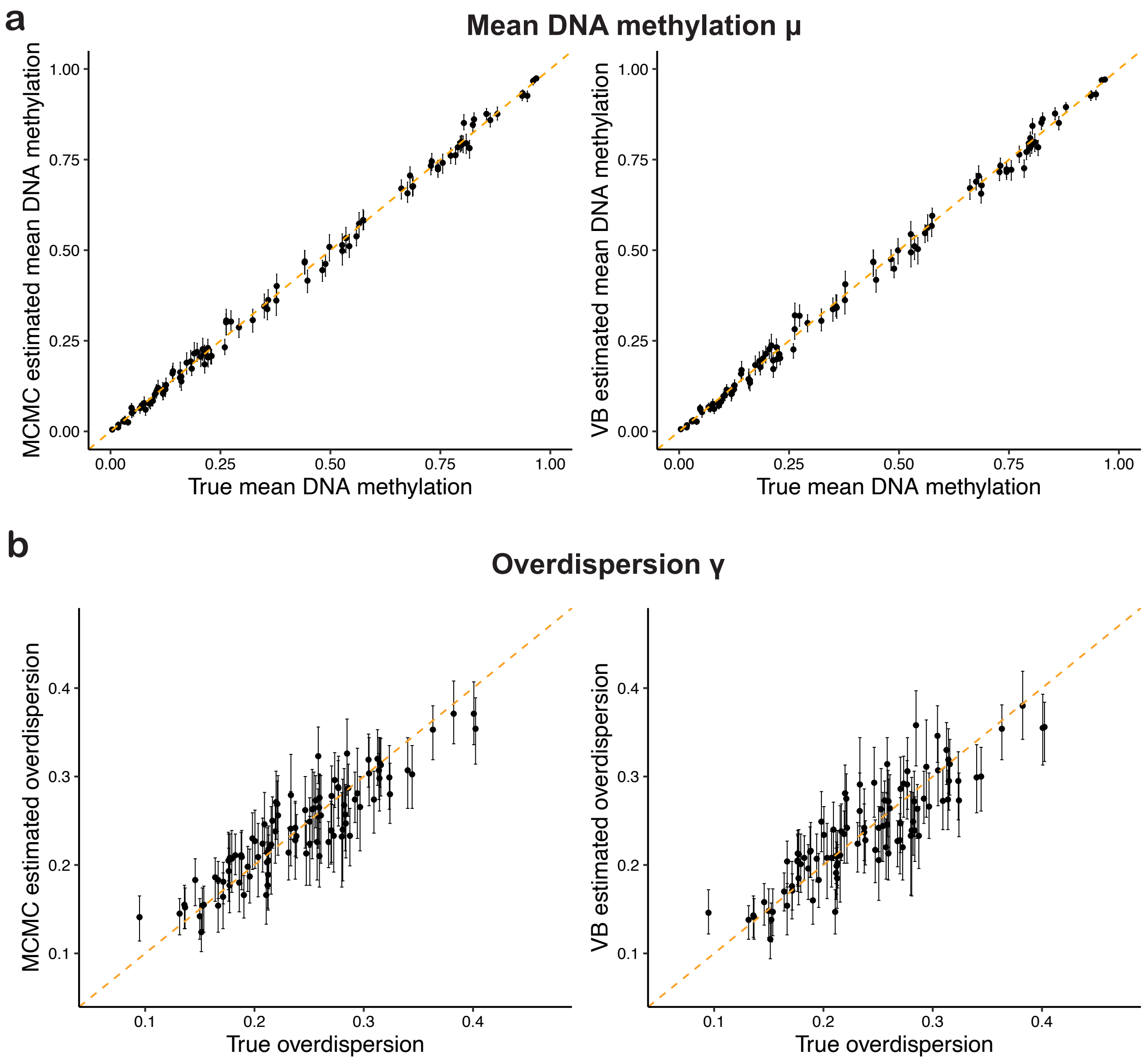

Figure S3: Markov Chain Monte Carlo (MCMC, left panels) and variational Bayes (VB, right panels) inference schemes display comparable estimation performance on simulated data for feature-specific (**a**) mean methylation μ_j and (**b**) overdispersion γ_j parameters. Dashed lines denote perfect agreement between true (x-axis) and estimated (y-axis) parameter values. Each data point represents a different features, dots show posterior medians and vertical lines correspond to 80% high posterior density (HPD) intervals, computed using the `coda` package (2).

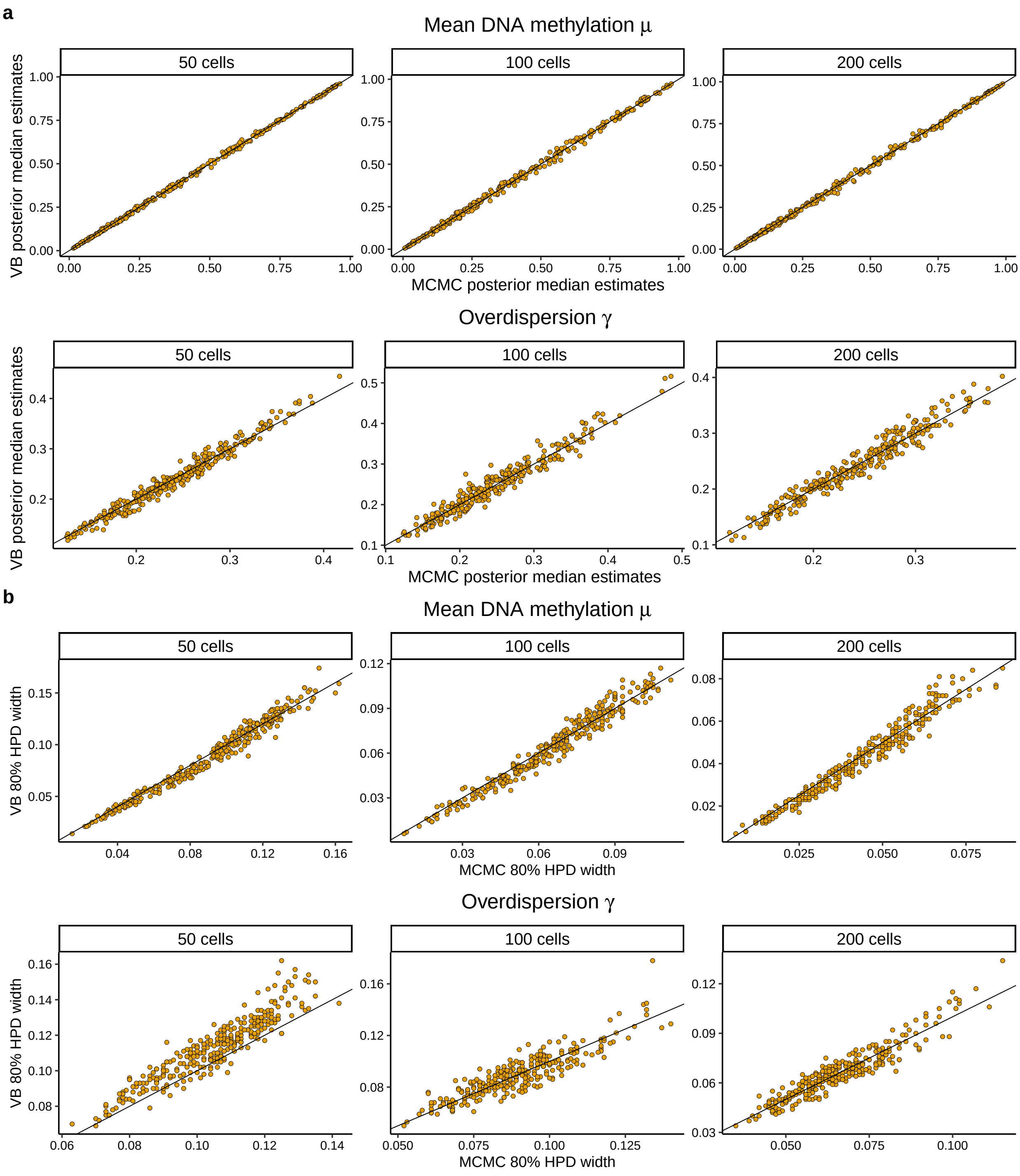

Figure S4: Markov Chain Monte Carlo (MCMC) and variational Bayes (VB) inference schemes infer similar posterior distributions for feature-specific μ_j and γ_j parameters. (**a**) Posterior medians using MCMC (x-axis) and VB (y-axis) for mean methylation (top) and overdispersion (bottom) parameters. (**b**) We compute the width of the 80% high posterior density (HPD) interval using MCMC (x-axis) and VB (y-axis) posterior draws for mean methylation (top) and overdispersion (bottom) parameters. Each data point represents a different feature.

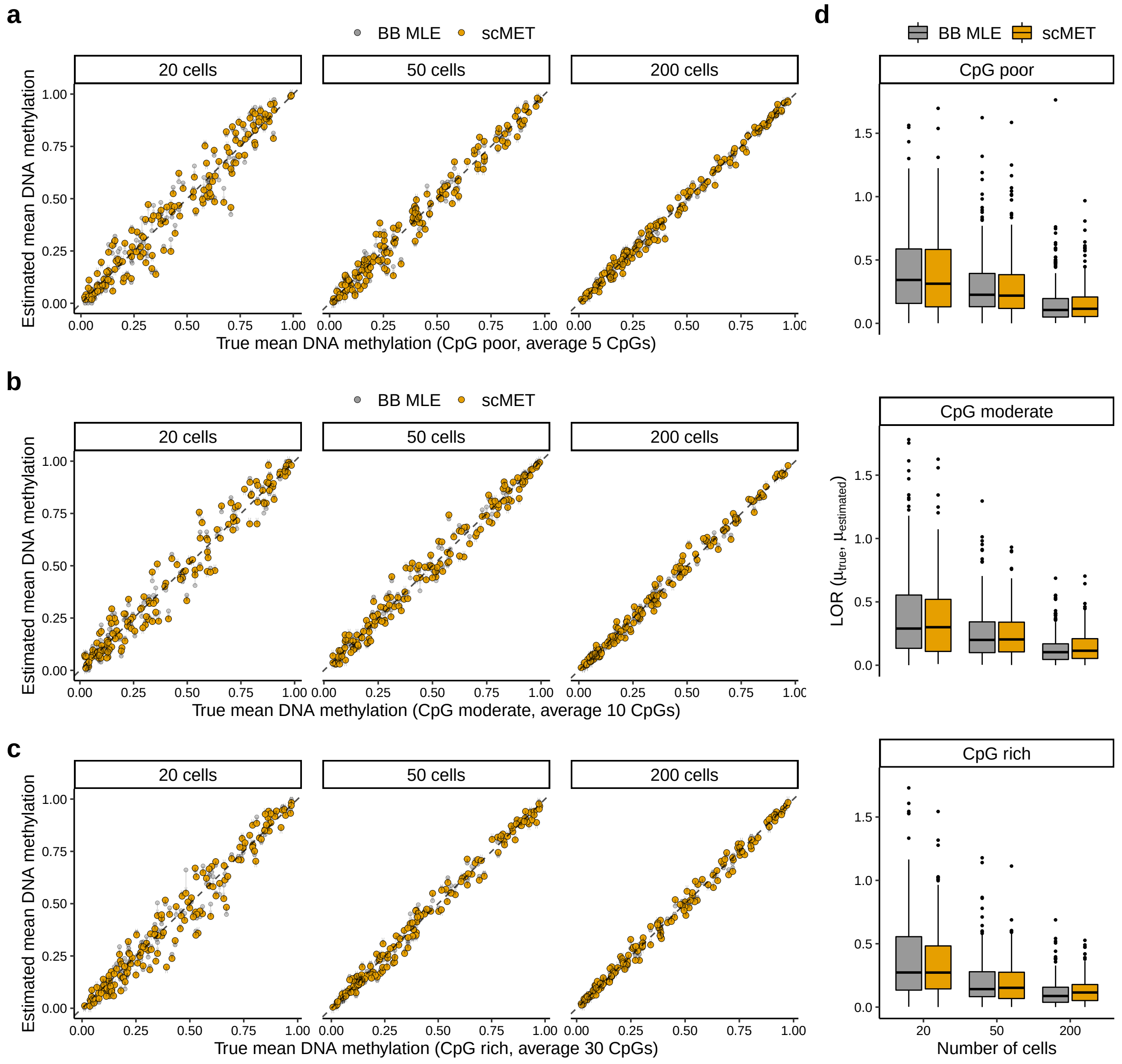

Figure S5: Shrinkage effect on posterior estimates for feature-specific mean methylation parameters μ_j. (**a, b, c**) Mean methylation estimates per feature for varying number of cells using the beta-binomial MLE (grey) and scMET (yellow) models. For scMET each data point represents the posterior median for the associated parameter. Vertical arrows denote the *shrinkage* that is introduced by scMET with respect to MLE estimates. The dashed line corresponds to perfect agreement between true (x-axis) and estimated (y-axis) mean methylation. As expected, the scMET and BB MLE estimates for μ_j parameters are comparable. (**a**) CpG poor regions, (**b**) CpG moderate regions, and (**c**) CpG rich regions. (**d**) For the synthetic data in (**a**), (**b**) and (**c**), we use the absolute log-odds ratio (LOR) difference between true and estimated values as a measure of estimation performance for varying number of cells. The smaller the LOR value the better the estimation performance. Each data point represents a feature.

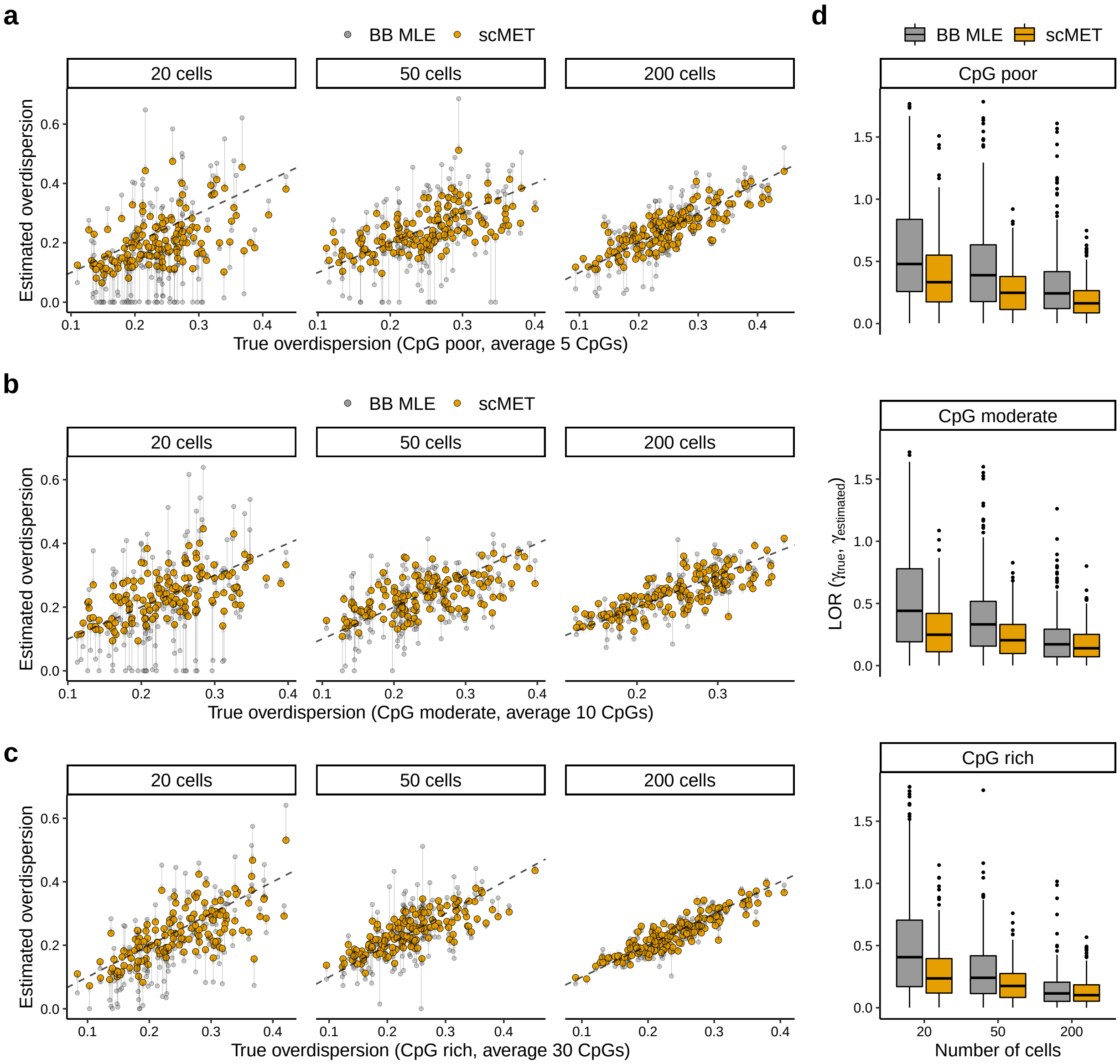

Figure S6: Shrinkage effect on posterior estimates for feature-specific overdispersion parameters γ_j. (**a, b, c**) Overdispersion estimates per feature for varying number of cells using the beta-binomial MLE (grey) and scMET (yellow) models. For scMET each point represents the posterior median for the associated parameter. Vertical arrows denote the *shrinkage* that is introduced by scMET with respect to MLE estimates. The dashed line corresponds to perfect agreement between true (x-axis) and estimated (y-axis) overdispersion. (**a**) CpG poor regions, (**b**) CpG moderate regions, and (**c**) CpG rich regions. (**c**) For the synthetic data in (**a**), (**b**) and (**c**), we use the absolute log-odds ratio (LOR) difference between true and estimated values as a measure of estimation performance for varying number of cells. The smaller the LOR value the better the estimation performance. Each data point represents a feature.

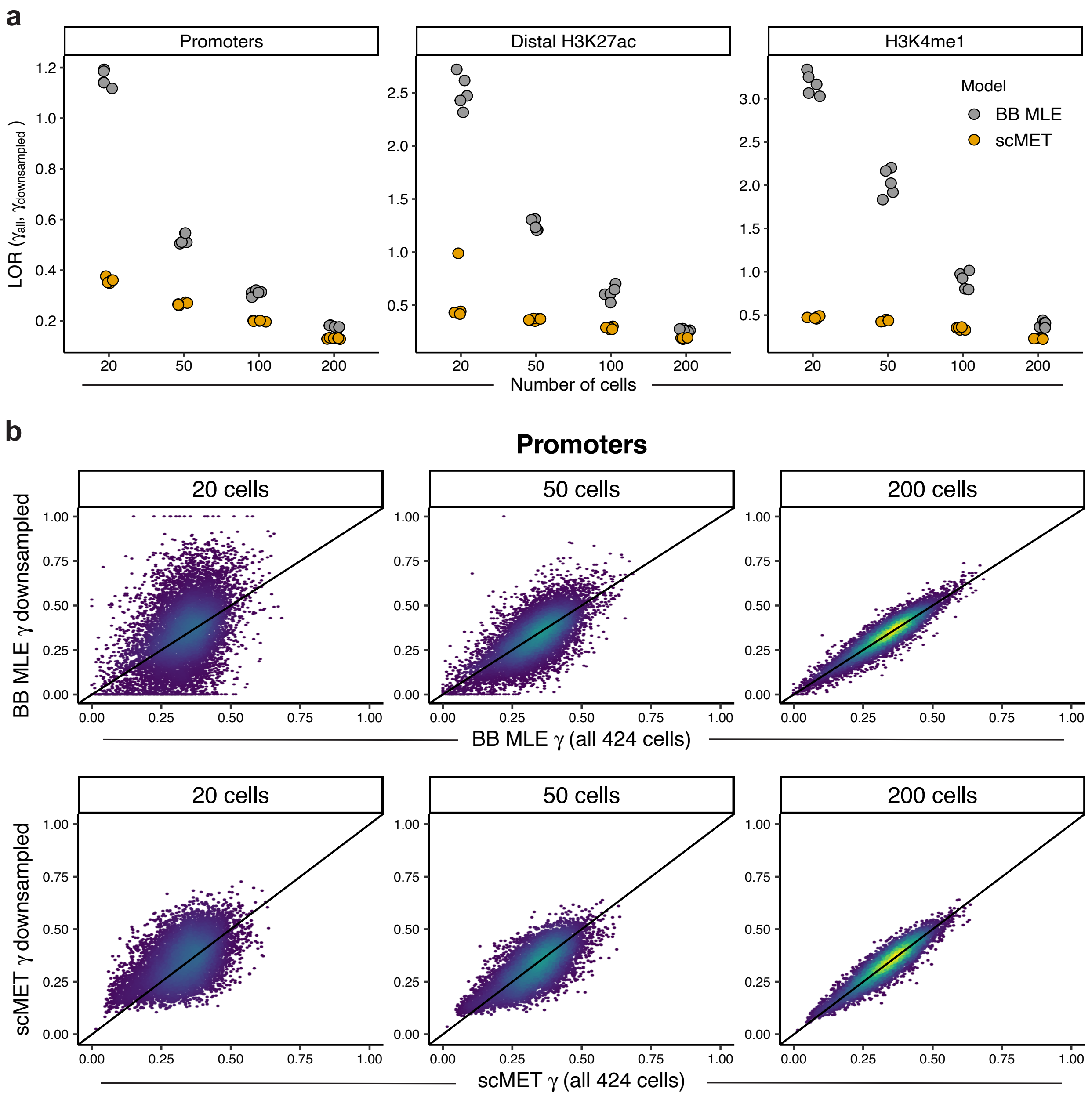

Figure S7: Down-sampling experiments to assess the performance of scMET (posterior medians) and BB MLE point estimates for feature-specific overdispersion parameters γ_j. In each down-sampling experiment a number of cells are randomly selected amongst the 424 inhibitory neurons characterised by (3). Estimates based on the full dataset are used as pseudo ground truth to assess the stability of inference for decreasing sample size. (**a**) Mean absolute log-odds ratio (LOR) difference as a measure of estimation performance for varying number of cells across the three genomic contexts considered in this study. The smaller the LOR value the better the estimation performance. Each data point represents a different random down-sampling experiment. (**b**) Estimates for feature-specific overdispersion parameters γ_j for varying number of cells using BB MLE (top) and scMET (bottom). Each facet compares point estimates of all 424 cells (x-axis) versus a single randomly down-sampled dataset (y-axis). Each data point corresponds to a different feature. The color code within the scatter-plots is used to represent areas with high (green and yellow) and low (blue) concentration of features. Promoter regions are shown as illustrative example; the remaining genomic contexts show a similar pattern (data not shown).

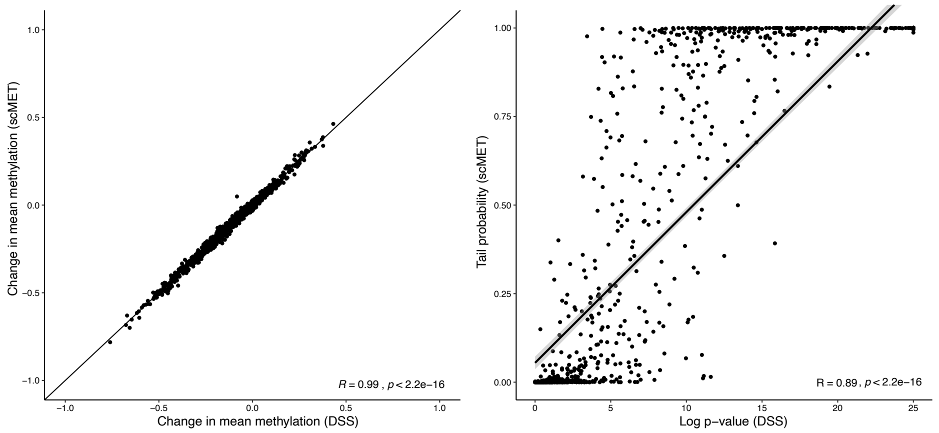

Figure S8: Comparing differential methylation estimates between scMET and DSS. (left) Comparing differential mean levels (point estimates) between DSS (x-axis) and scMET (y-axis). Shown is the y=x line. (right) Comparing log p-values inferred by DSS (x-axis) with differential tail probabilities inferred by scMET (y-axis).

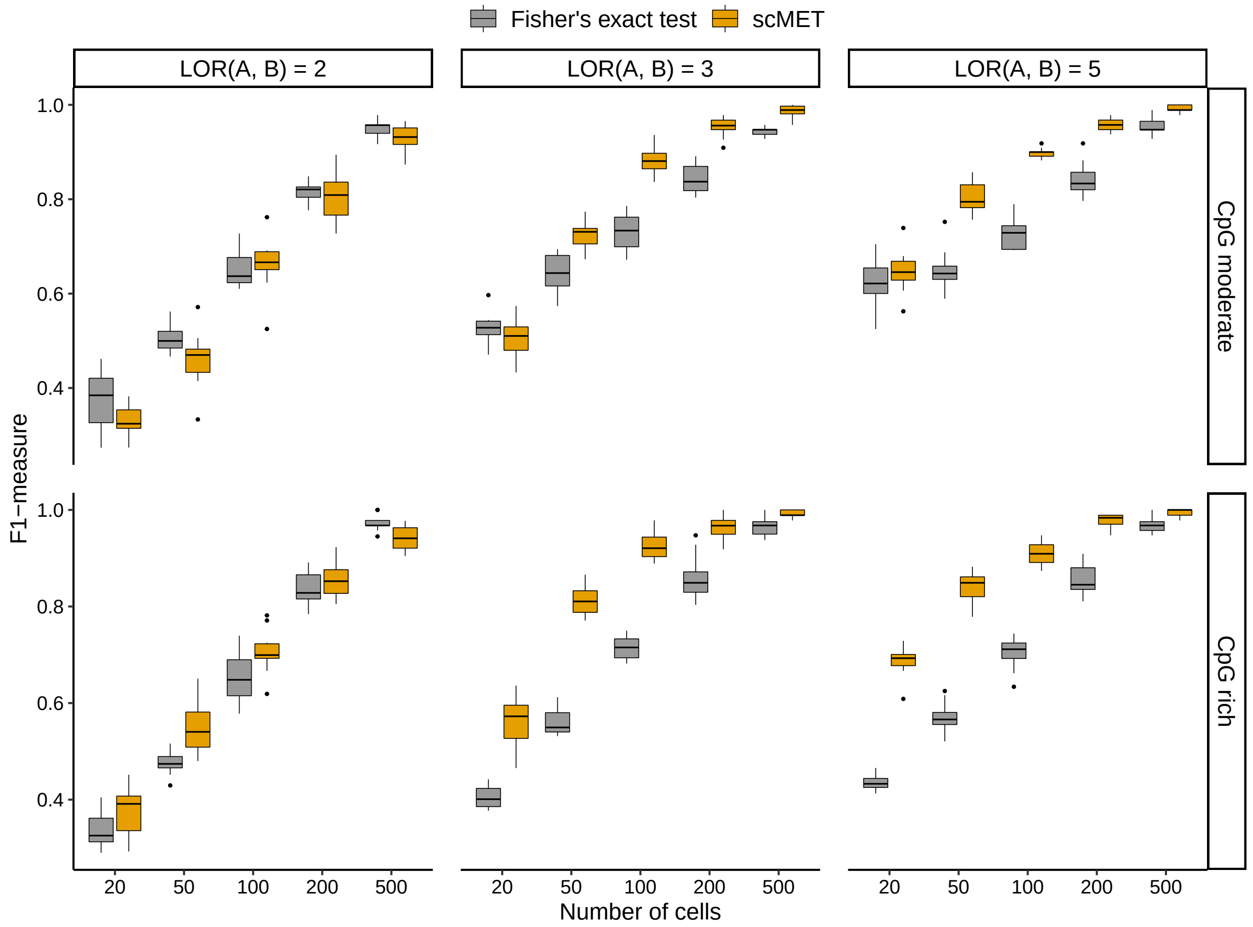

Figure S9: Performance for the differential mean methylation test in terms of F1-measure. We compare hits obtained by scMET (yellow) and Fisher's exact test (grey) in accurately identifying differentially methylated features for varying number of cells (x-axis) and across different settings. Column facets (boxes) correspond to simulating differentially methylated features with different effect sizes, in terms of log-odds ratio (LOR). Row facets correspond to simulated datasets with CpG moderate (on average 10 CpGs) and CpG rich (on average 30 CpGs) genomic regions (*Methods*). Each data point represents a different synthetic dataset.

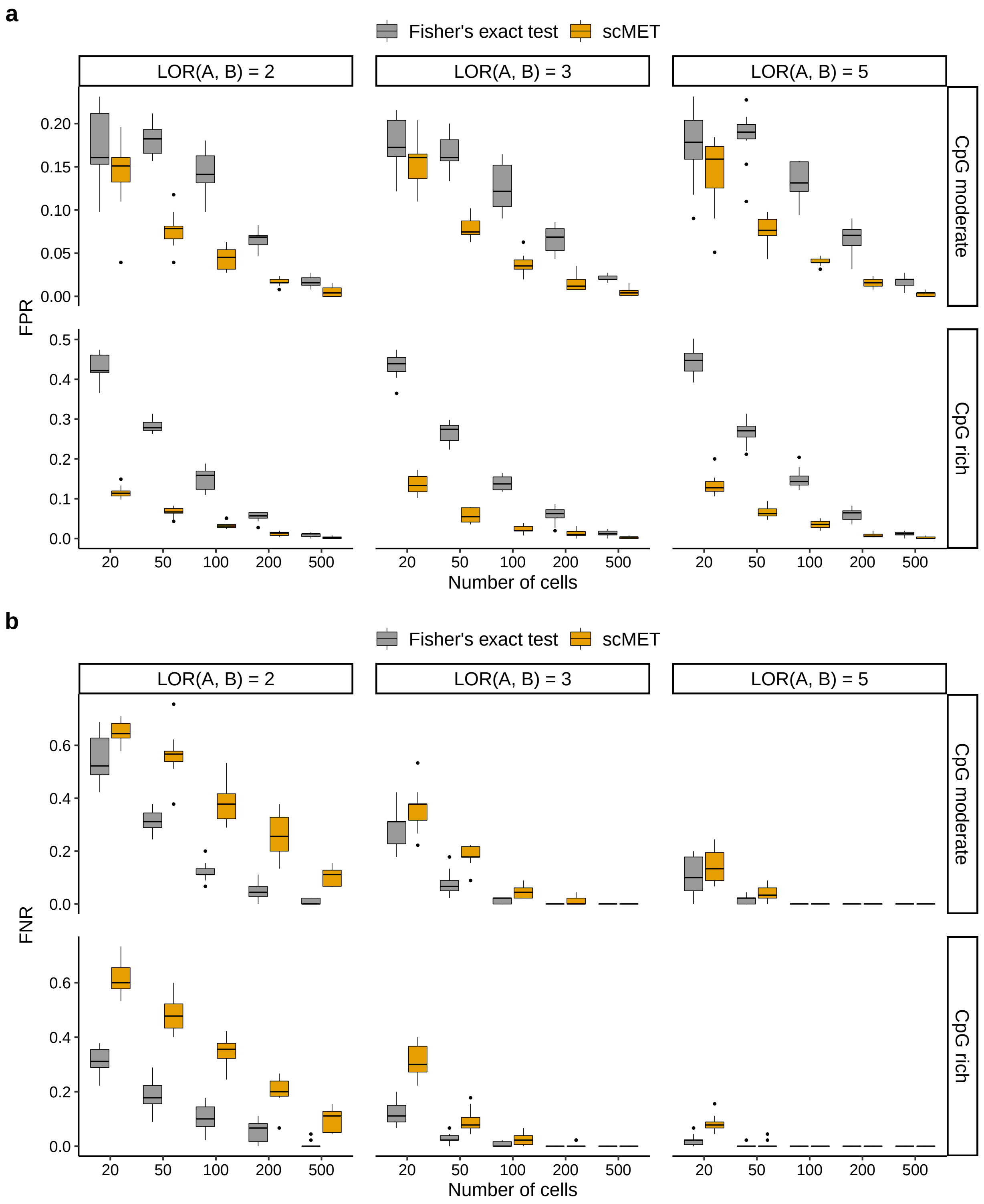

Figure S10: Performance for the differential mean methylation test in terms of false positive rate (FPR, **a**) and false negative rate (FNR, **b**). We compare hits obtained by scMET (yellow) and Fisher's exact test (grey) in accurately identifying differentially methylated features for varying number of cells (x-axis) and across different settings. Column facets (boxes) correspond to simulating differentially methylated features with different effect sizes, in terms of log-odds ratio (LOR). Row facets correspond to simulated datasets with CpG moderate (on average 10 CpGs) and CpG rich (on average 30 CpGs) genomic regions (*Methods*). Each data point represents a different synthetic experiment.

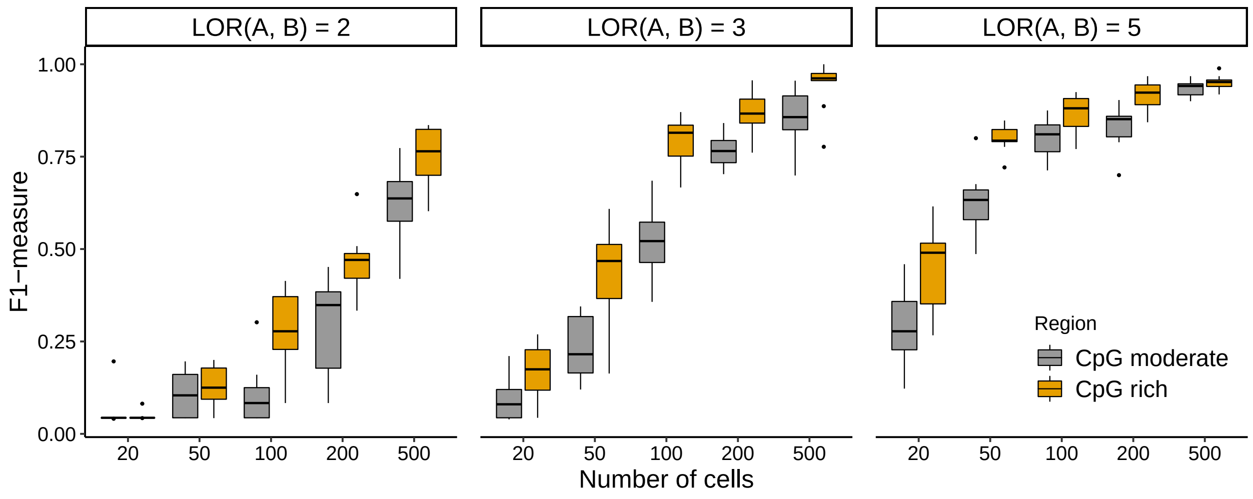

Figure S11: Performance for the differential variability test in terms of F1-measure (y-axis) for varying number of cells (x-axis). Colours correspond to simulated datasets with CpG moderate (grey, on average 10 CpGs) and CpG rich (yellow, on average 30 CpGs) genomic regions (*Methods*). Column facets (boxes) correspond to simulating differentially variable features with different effect sizes, in terms of log-odds ratio (LOR). Each data point represents a different synthetic experiment.

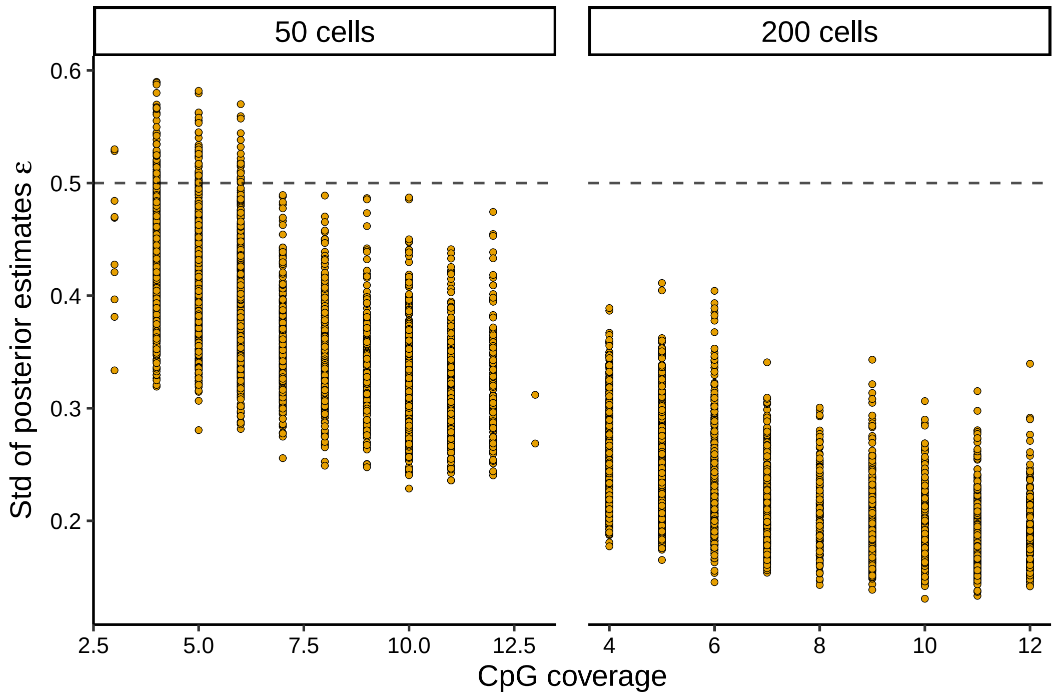

Figure S12: The average number of CpGs (CpG coverage) per feature (x-axis) is plotted against the posterior standard deviation for residual overdispersion ϵ_j (y-axis), when simulating 50 (left sub-panel) and 200 (right sub-panel) cells. These results were obtained using synthetic data, as described in *Methods*.

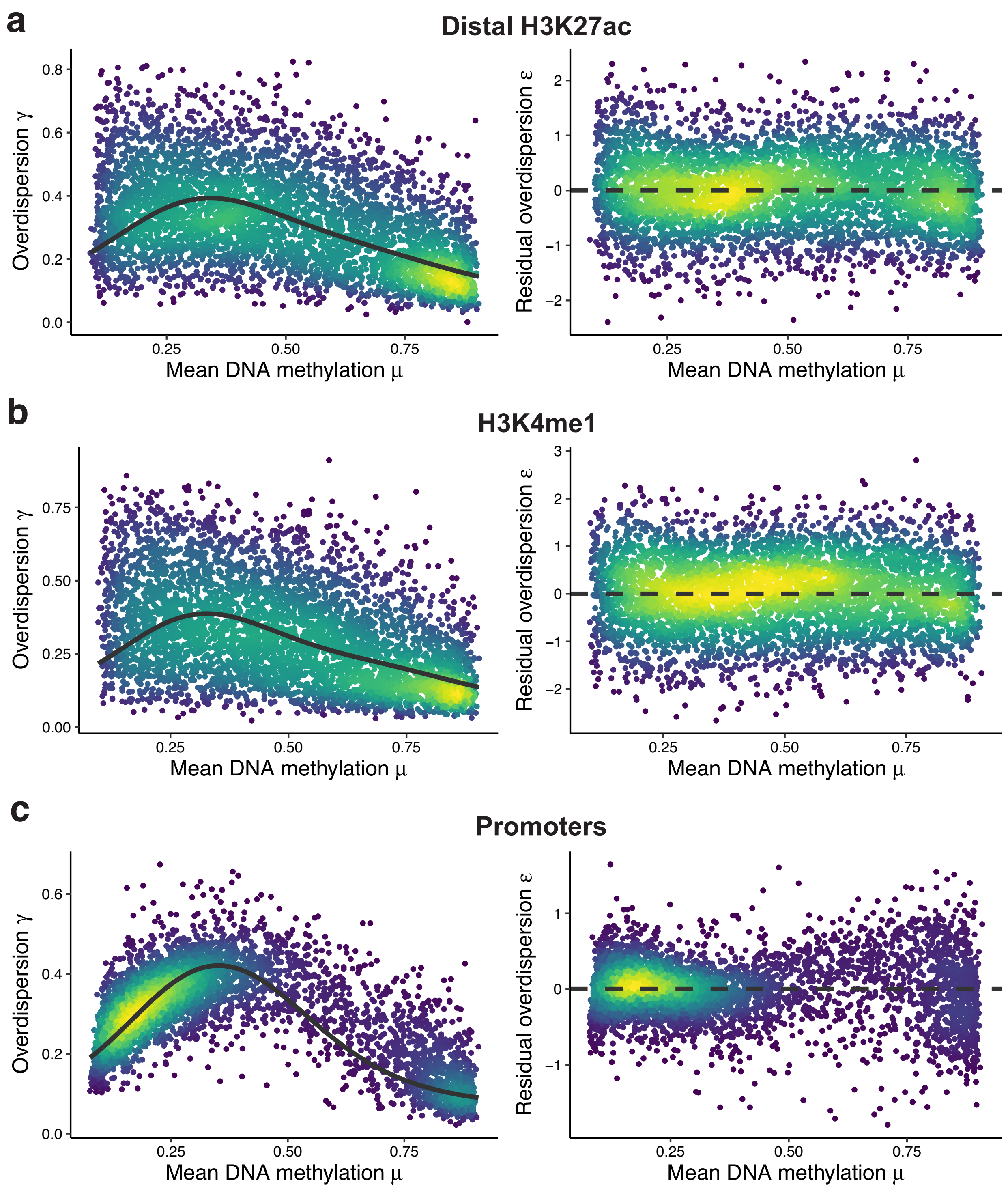

Figure S13: Mean-overdispersion relationship across genomic contexts for the (3) dataset. Each horizontal subpanel corresponds to a different genomic context. Left panels show posterior medians for feature-specific mean and overdispersion parameters. Black line represents the estimated regression trend from the GLM component of scMET (see Fig. 1a). Right panels show posterior medians for feature-specific methylation parameters μ_j (x-axis) versus residual overdispersion parameters ϵ_j (y-axis). Each data point corresponds to a different feature. The color code within the scatter-plots is used to represent areas with high (green and yellow) and low (blue) concentration of features.

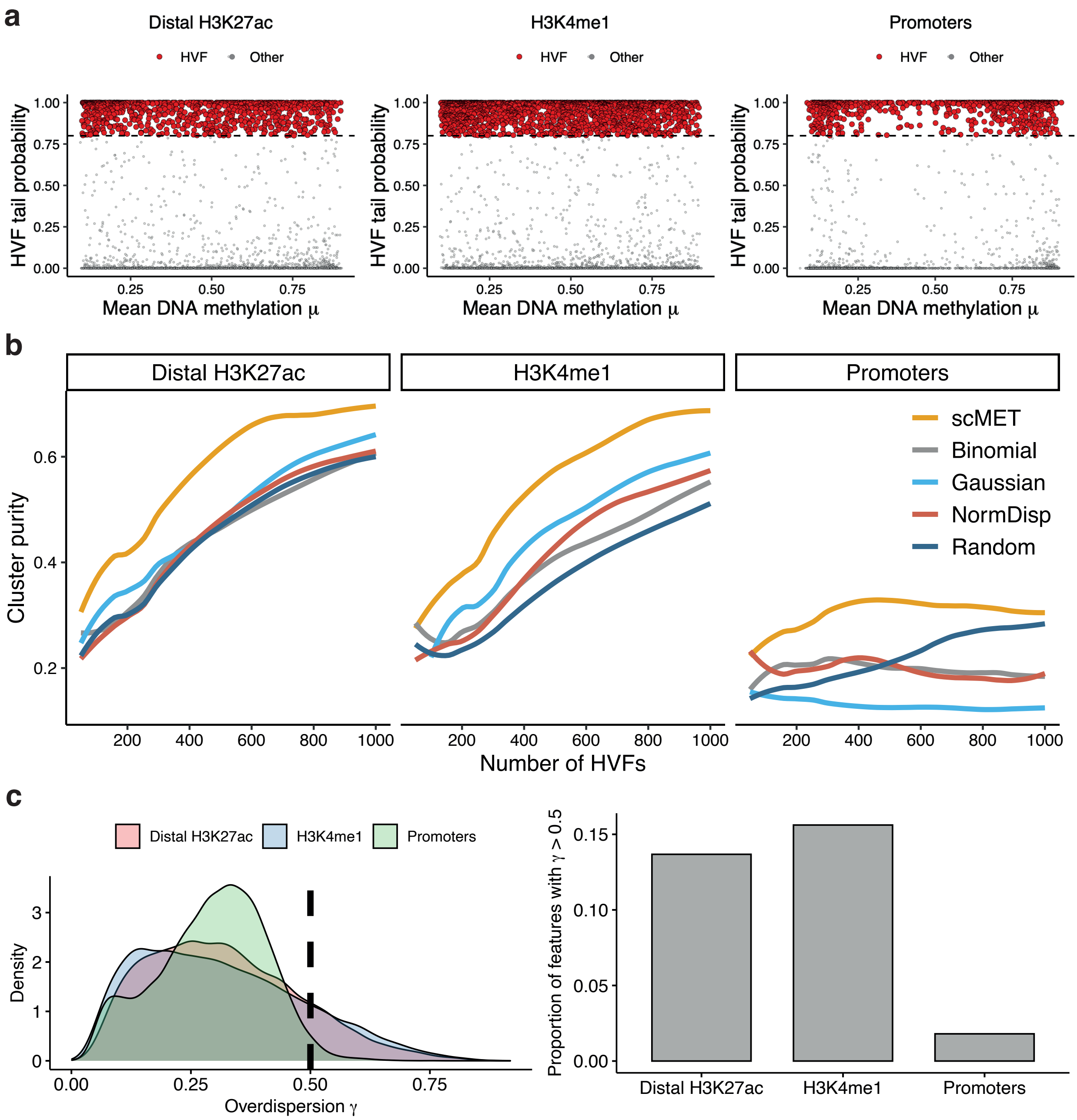

Figure S14: Identifying highly variable features (HVFs). (**a**) scMET tail posterior probabilities denoting the evidence of a feature being called as HVF. Each panel corresponds to a different genomic context for the (3) dataset. Red points correspond to features that are labelled as HVF. The horizontal dashed line corresponds to the posterior evidence threshold α_H set to match a desired EFDR = 10% (*Methods*). (**b**) Clustering performance, in terms of cluster purity (see Section S2.4), for varying number of selected HVFs. HVF selection was based on scMET (yellow), binomial variance (grey) and Gaussian variance (blue). A finite grid of HVFs was used for cluster purity evaluation and non-parametric regression was used to obtain a smoothed interpolation across all values (*Methods*) (**c**) Distribution of posterior estimates for feature-specific overdispersion parameters γ_j across different genomic contexts (left). Percentage of features with $\gamma_j > 0.5$ per genomic context (right).

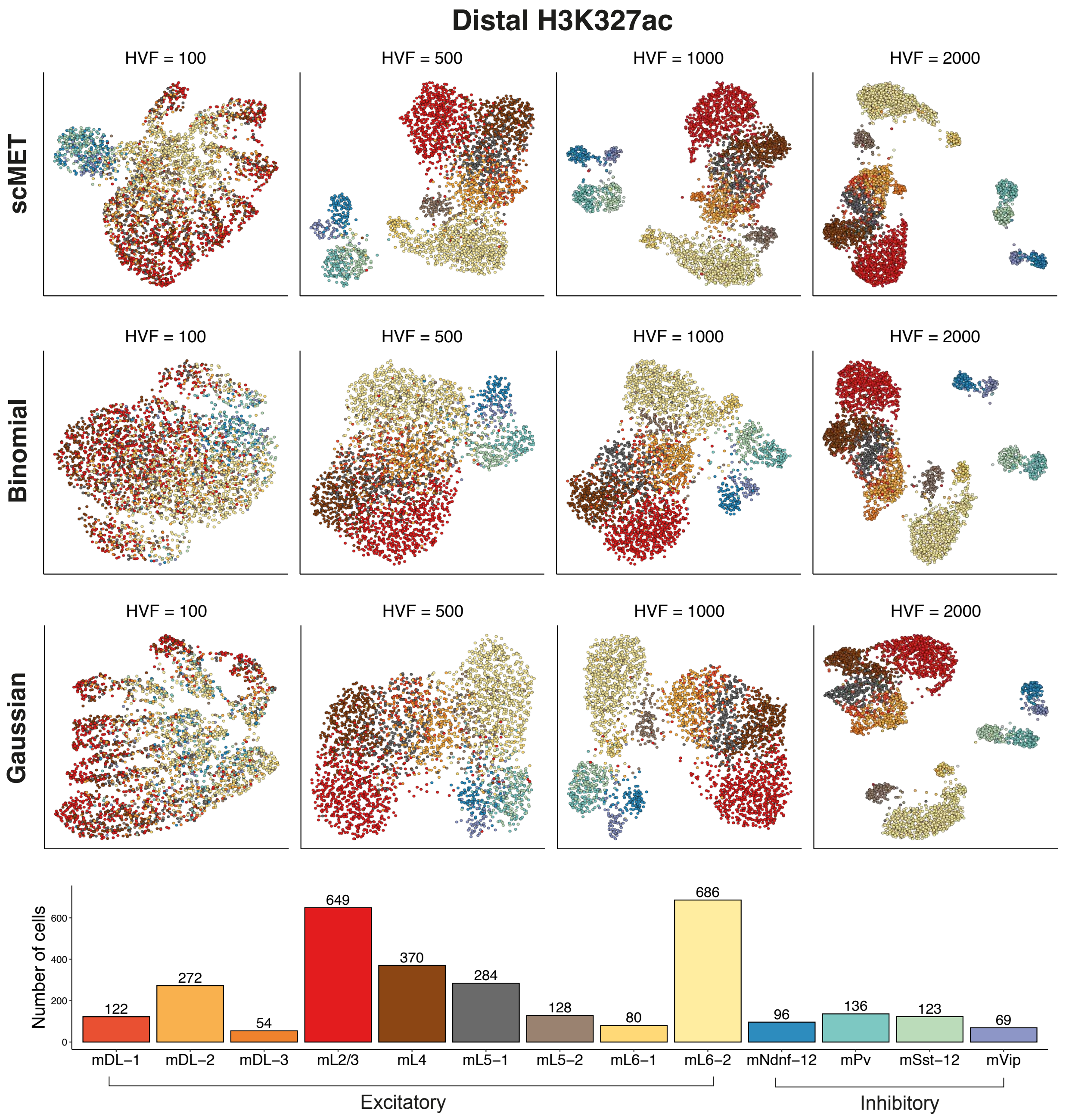

Figure S15: UMAP representations for varying number of HVFs when applied to distal H3K27ac features on the (3) dataset. Each row corresponds to a different method for HVF selection (scMET, Binomial and Gaussian). Each column corresponds to different number of HVFs. Each data point corresponds to a different cell. Points are coloured according to cell type assignment in the (3) study as shown in the bottom panel.

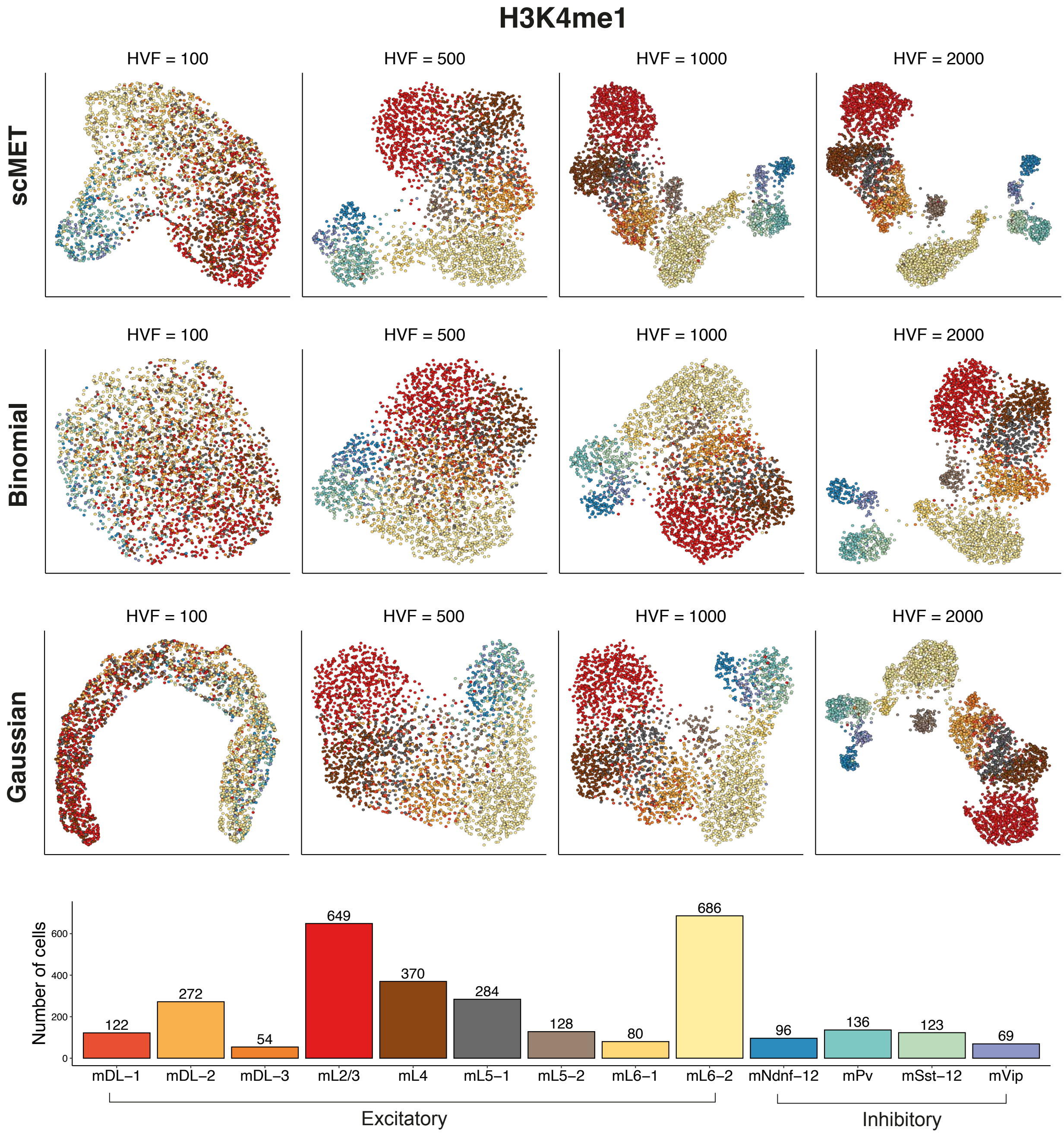

Figure S16: UMAP representations for varying number of HVFs when applied to H3K4me1 features on the (3) dataset. Each row corresponds to a different method for HVF selection (scMET, Binomial and Gaussian). Each column corresponds to different number of HVFs. Each data point corresponds to a different cell. Points are coloured according to cell type assignment in the (3) study as shown in the bottom panel.

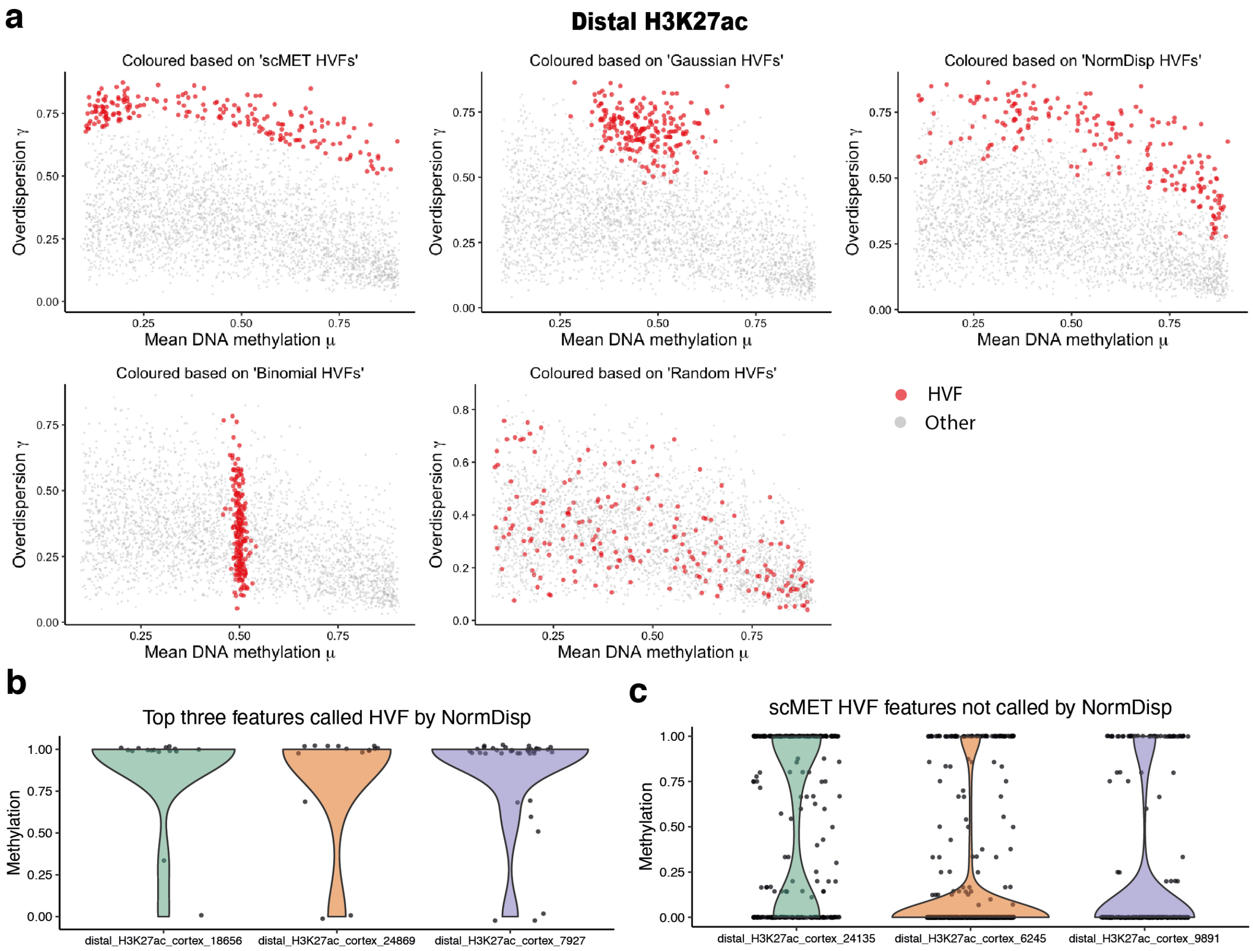

Figure S17: (**a**) Mean-overdispersion relationship for the distal H3K27ac genomic context, as estimated by scMET. Red points correspond to the top 200 HVFs being called by the corresponding method given in the title of each sub-panel. (**b**) Top three HVFs, based on highest normalised dispersion value, as computed by the NormDisp method. These features were not called as HVF by scMET. (**c**) Top three HVFs (that were not called by NormDisp) based on scMET's residual overdispersion.

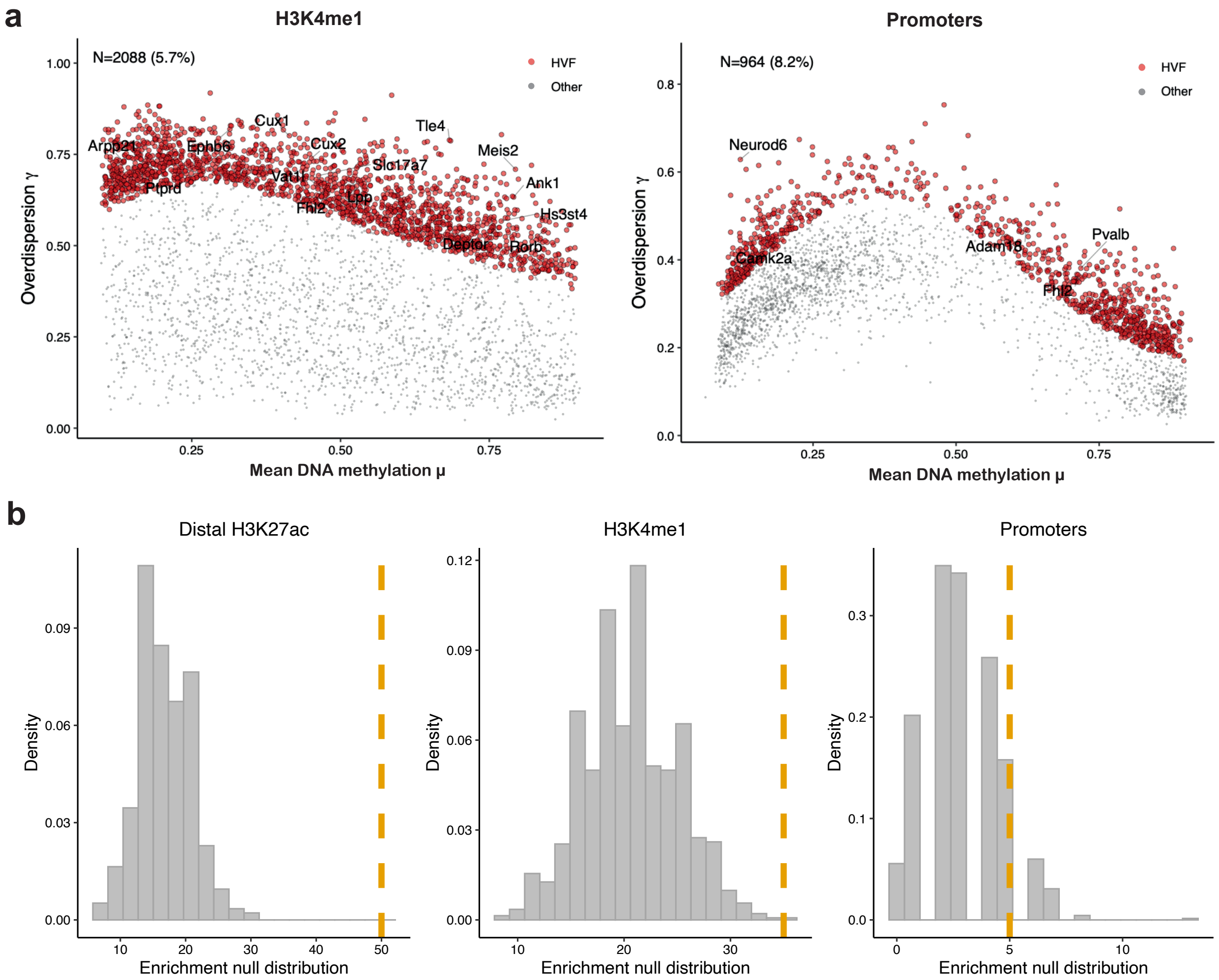

Figure S18: Identifying highly variable features (HVFs) on the (3) dataset. (**a**) Mean-overdispersion plots for H3K4me1 and promoter genomic contexts. Red points correspond to features being called as HVF (EFDR = 10% and percentile threshold $\delta_E = 90\%$). To ease interpretation each element is linked to its nearest gene. (**b**) Histogram showing enrichment distribution under the null model. The null model is based on randomly labelling features as HVFs and then counting how many of those are overlapping with neuron marker genes identified by (3), see Supplementary Table S1. To obtain a null distribution we repeated this process 1,000 times. The dashed vertical yellow line, shows the enrichment of HVFs using the scMET model.

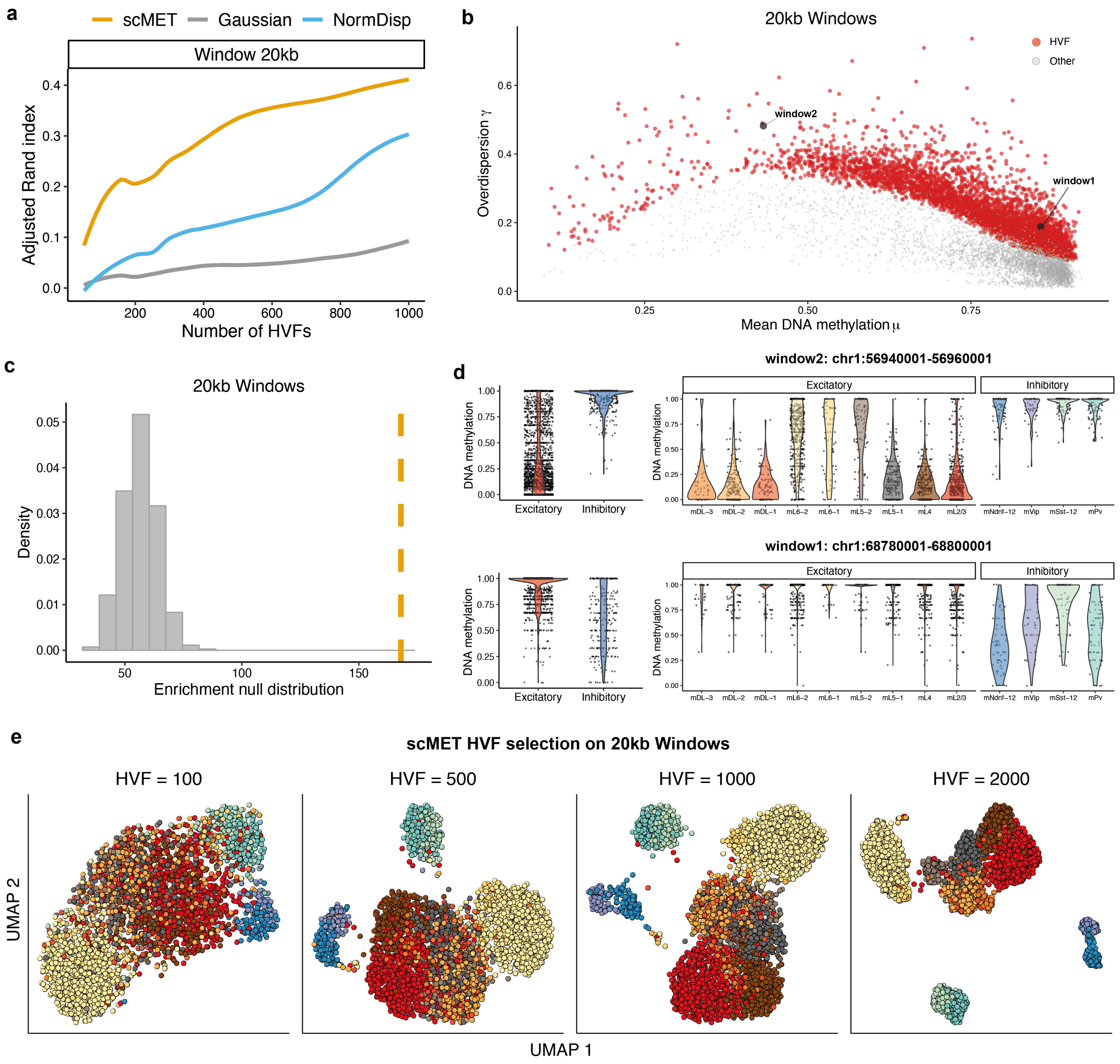

Figure S19: Identifying genome-wide HVFs from 20kb sliding-windows in the mouse brain cortex dataset. (**a**) Clustering performance in terms of adjusted Rand index (ARI) for varying number of HVFs. HVF selection was based on scMET's residual overdispersion parameters ϵ_j (yellow), Gaussian variance (grey) and normalised dispersion (NormDisp, cyan). A finite grid of HVFs was used for ARI evaluation and non-parametric regression was used to obtain a smoothed interpolation across all values. (**b**) Mean methylation (μ_j, x-axis) is plotted against overdispersion estimates (γ_j, y-axis). Red points correspond to features being called as HVF by scMET. (**c**) Histogram showing enrichment distribution under the null model. The null model is based on randomly labelling features as HVFs and then counting how many of those are overlapping with neuron marker genes identified by (3), see Supplementary Table S1. To obtain a null distribution we repeated this process 1,000 times. The dashed vertical yellow line, shows the enrichment of HVFs using the scMET model. (**d**) Examples of windows with large residual overdispersion which overlap with *Satb2* (top) a marker gene for excitatory neurons, and *Erbb4* (bottom) a marker gene of inhibitory neurons. Left sub-panels stratify cells by the broad neuronal class. Right sub-panels group cells by the neuronal subclass. (**e**) UMAP representation for varying number of HVFs when applied to 20kb sliding windows on the mouse brain cortex dataset. scMET was run independently per chromosome. Top HVF features were subsequently identified on the genome-wide dataset based on residual overdispersion parameters ϵ_j (see *Methods).*

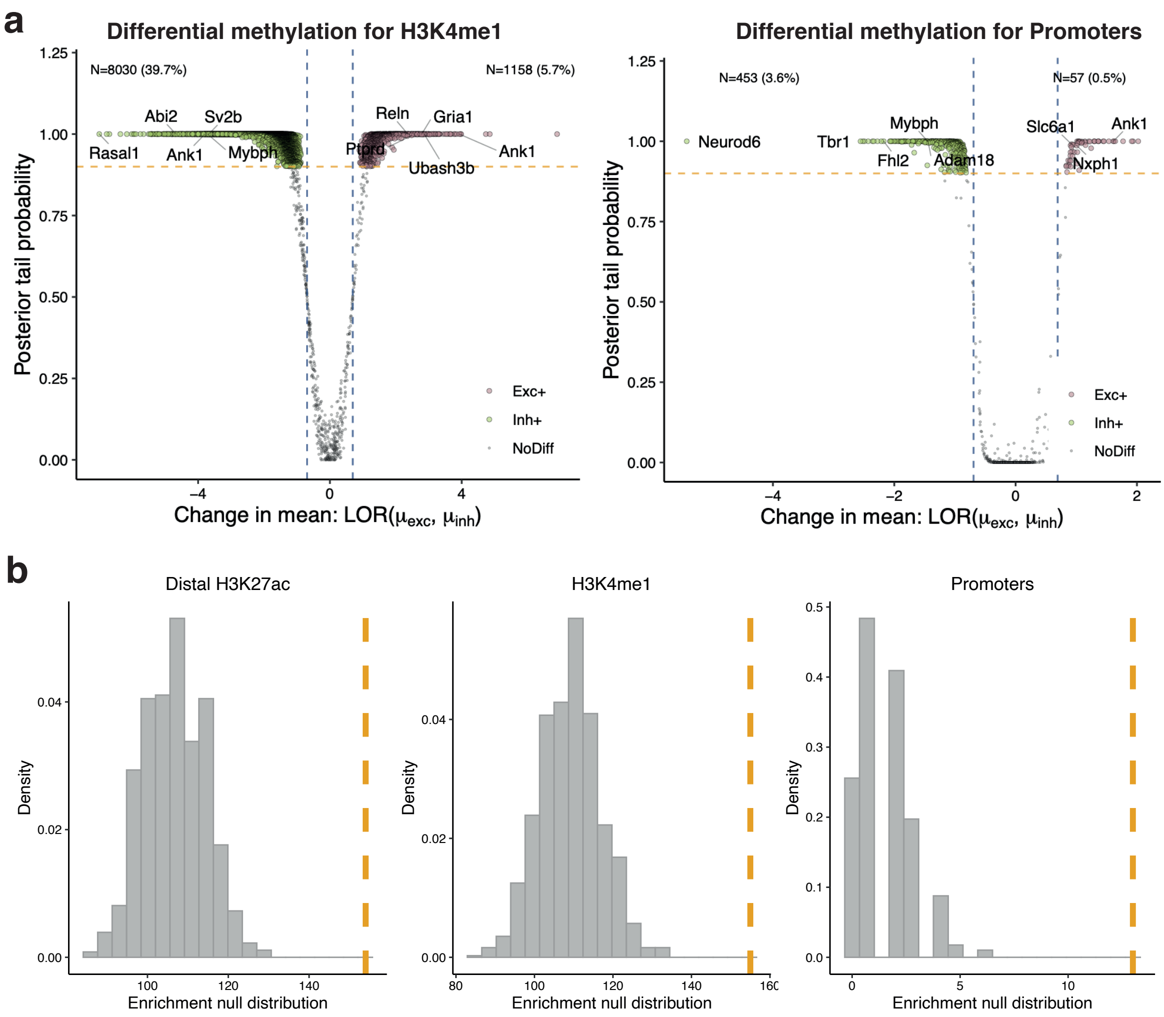

Figure S20: Identifying differentially methylated (DM) features across excitatory and inhibitory neurons on the (3) dataset. (**a**) Volcano plots of DM features for H3K4me1 and promoter genomic contexts. (**b**) Histogram showing enrichment distribution under the null model. The null model is based on randomly labelling features as DM and then counting how many of those are overlapping with neuron marker genes identified in (3), see Supplementary Table S1. To obtain a null distribution we repeated this process 1,000 times. The dashed vertical yellow line, shows the enrichment of DM features using the scMET model.

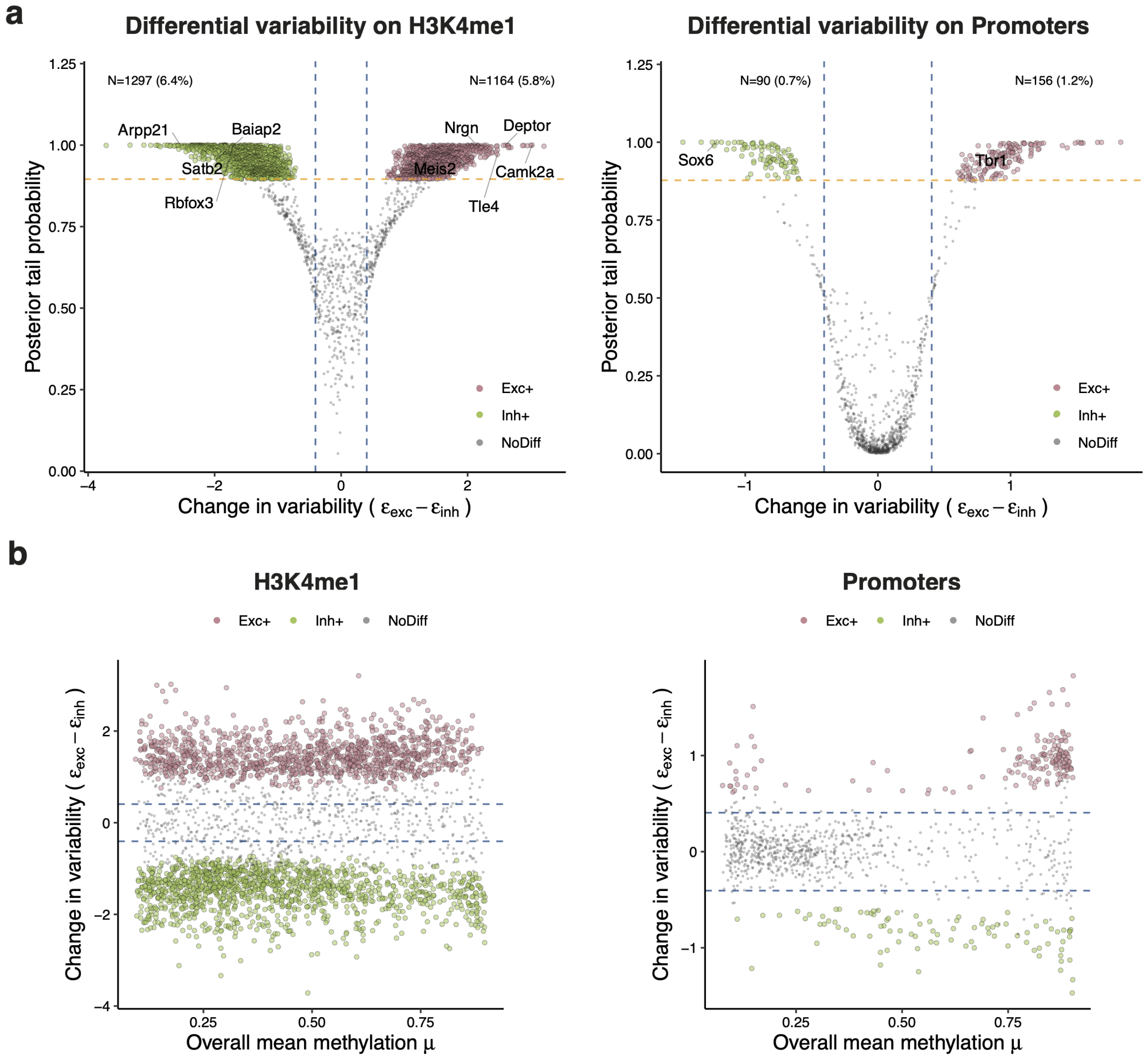

Figure S21: Identifying differentially variable (DV) features across excitatory and inhibitory neurons on the (3) dataset. (**a**) Volcano plots of DV features for H3K4me1 and promoter genomic contexts. (**b**) For each feature, the overall mean methylation across all cells is plotted against the change in residual overdispersion between excitatory and inhibitory neurons. Features with statistically significant changes in variability are coloured according to their regulation. Red-like and green-like colours denote features with higher variability in excitatory and inhibitory neurons, respectively.

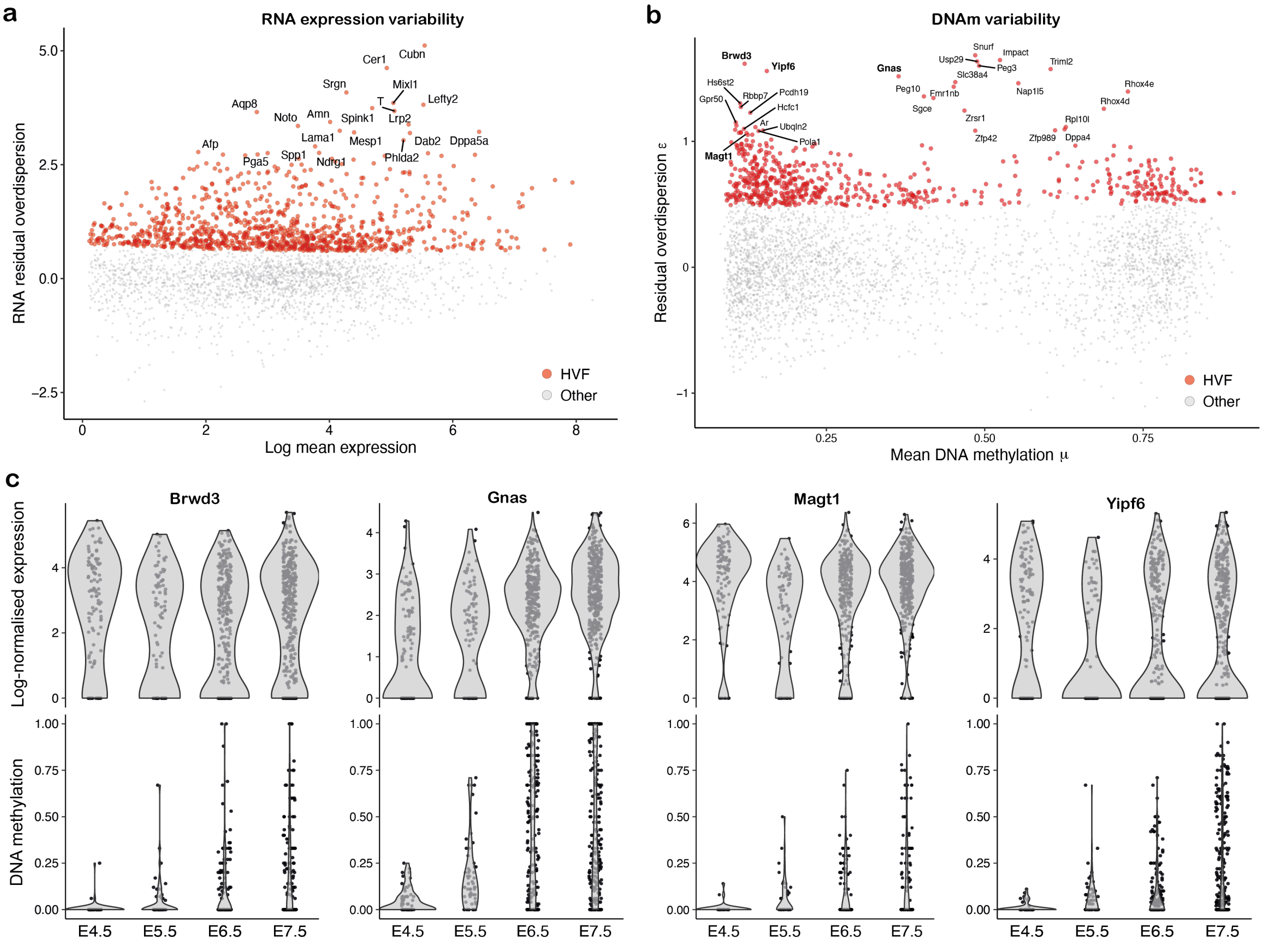

Figure S22: Exploring the relationship between transcriptional and DNAm variability on the multi-omics scNMT-seq gastrulation dataset (4). Mean (x-axis) versus residual overdispersion estimates (y-axis) for (**a**) RNA expression and (**b**) promoter DNA methylation modalities for the scNMT-seq gastrulation dataset. (**c**) Representative examples of the DNAm and RNA expression patterns for genes with high DNA methylation heterogeneity and low RNA expression heterogeneity. Y-axis shows BASiCS log-normalised gene expression (in a $\log(x+1)$ scale) (top) and promoter DNAm rate (bottom). Cells are stratified by embryonic stage (x-axis).

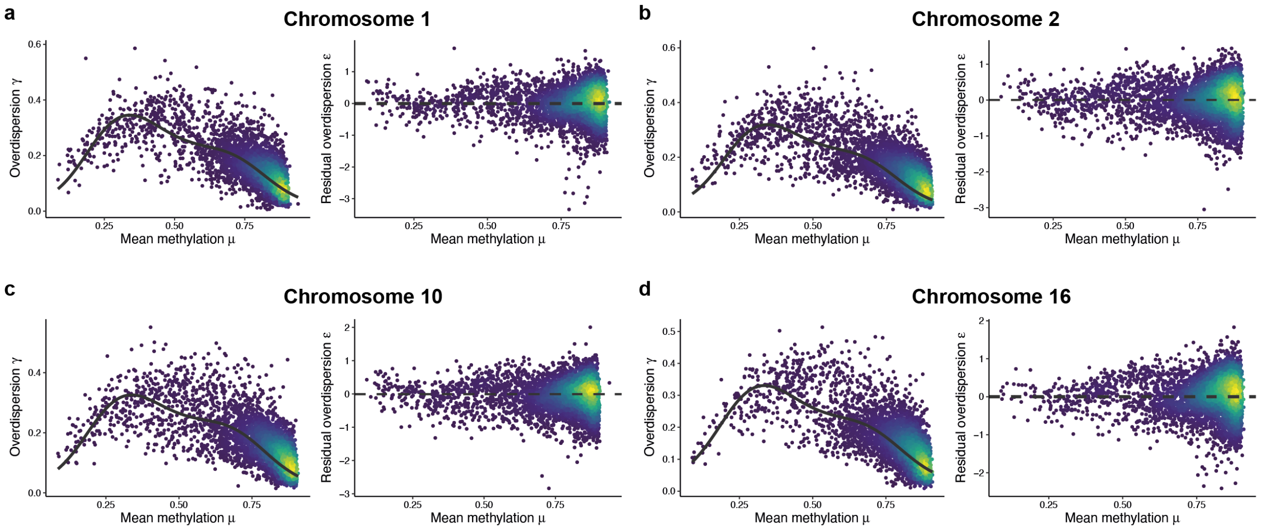

Figure S23: Mean-overdispersion relationship for the mouse brain cortex dataset, across four different chromosomes. Left sub-panels show posterior medians for feature-specific mean (μ_j, x-axis) and overdispersion (γ_j, y-axis) parameters. Black line represents the estimated regression trend from the GLM component of scMET. Right sub-panels show posterior medians for feature-specific methylation parameters (μ_j, x-axis) versus residual overdispersion parameters (ϵ_j, y-axis). Each data point corresponds to a different feature. The color code within the scatter-plots is used to represent areas with high (green and yellow) and low (blue) concentration of features. For simplicity we show 4 representative examples, but similar trends are observed for the remaining chromosomes.

S2 Supplementary notes

S2.1 Choice of a beta-binomial (BB) likelihood

The BB model has been successfully applied in the context of bulk DNA methylation data (5); therefore constitutes a natural building block when modelling single cell DNA methylation measurements. Here, we provide a brief description to motivate its use.

In a single-cell bisulphite assay, the output is a binary readout (methylated or unmethylated) for each cell i and CpG site j. This can be modelled with a Bernoulli distribution. However, it is often preferred to pool information across CpG sites and instead work with predefined regions of several base pairs long (e.g. sliding windows, promoters or enhancers). Here, we refer to these regions as features. For each feature j and cell i, the number of methylated CpGs (Y_{ij}) can be modelled as:

$$Y_{ij}|\theta_j \sim \text{Binomial}(n_{ij}, \mu_j), \tag{1}$$

where n_{ij} represent the coverage for each cell and region. Within a region, this assumes that CpGs are independent and have the same probability of being methylated (μ_j), which is constant across all cells. However, single cell methylation data typically exhibits more variability than what is predicted by this model. This is due to the presence of biological variability, e.g. multiple cell types in the population. This additional variability can be captured using the BB model, defined as:

$$Y_{ij}|\theta_{ij} \sim \text{Binomial}(n_{ij}, \theta_{ij}) \tag{2}$$

$$\theta_{ij}|\mu_j, \gamma_j \sim \text{Beta}(\mu_j, \gamma_j), \tag{3}$$

where γ_j is an overdispersion parameter that ranges from 0 to 1. If $\gamma_j = 0$ (no biological variability), this model reduces to the one in equation (1).

S2.2 scMET prior specification

To complete the scMET model we introduce the following priors for the remaining parameters,

$$\mathbf{w}_\mu \sim \text{MVN}(\mathbf{m}_{w\mu}, s_{w\mu}\mathbf{I}), \quad \mathbf{w}_\gamma \sim \text{MVN}(\mathbf{m}_{w\gamma}, s_{w\gamma}\mathbf{I}), \quad s_\gamma \sim \text{IG}(\alpha_{s\gamma}, \beta_{s\gamma}),$$

where MVN($\cdot$) denotes the multivariate-normal distribution, IG($\cdot$) the inverse-gamma distribution, and $\mathbf{I}$ the identity matrix with appropriate dimensions. The joint distribution over the observed and latent variables for the scMET model is given by,

$$\begin{aligned} p(\mathbf{Y}, \boldsymbol{\mu}, \boldsymbol{\gamma}, \mathbf{w}_\mu, s_\mu, \mathbf{w}_\gamma, s_\gamma \mid \mathbf{X}) = p(\mathbf{Y} \mid \boldsymbol{\mu}, \boldsymbol{\gamma})\; p(\boldsymbol{\mu} \mid \mathbf{w}_\mu, s_\mu, \mathbf{X}) \\ p(\boldsymbol{\gamma} \mid \boldsymbol{\mu}, \mathbf{w}_\gamma, s_\gamma)\; p(\mathbf{w}_\mu)\; p(\mathbf{w}_\gamma)\; p(s_\gamma), \end{aligned} \tag{4}$$

where the factorisation corresponds to the probabilistic graphical model provided in Fig. 1a and Supplementary Fig. S24.

S2.2.1 Choice of hyper-parameters

For all experiments performed in this study the following hyper-parameters were fixed *a priori* to the following default values:

$$s_\mu = 1.5, \quad s_{w\mu} = 2, \quad s_{w\gamma} = 2, \quad \alpha_{s\gamma} = 2, \quad \beta_{s\gamma} = 3$$

For the $\mathbf{m}_{w\mu}$ and $\mathbf{m}_{w\gamma}$ hyper-parameters we employed an empirical Bayes approach (6) to set default values based on the data. To do so, we first obtained MLE estimates $\hat{\mu}_j$ and $\hat{\gamma}_j$ using the

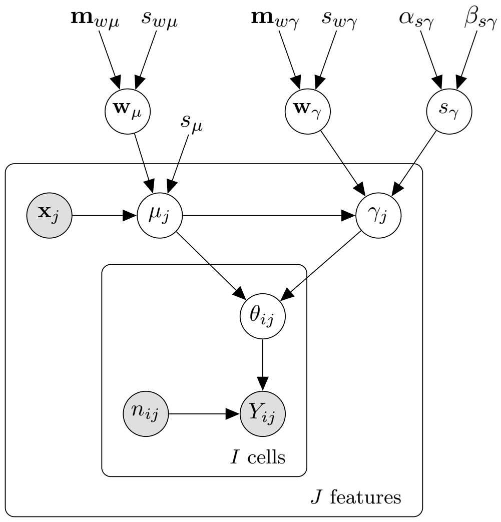

$$
\begin{aligned}
s_\gamma &\sim \text{IG}(\alpha_{s\gamma}, \beta_{s\gamma}) \\
\mathbf{w}_\gamma &\sim \mathcal{N}(\mathbf{m}_{w\gamma}, s_{w\gamma}) \\
\mathbf{w}_\mu &\sim \mathcal{N}(\mathbf{m}_{w\mu}, s_{w\mu}) \\
\gamma_j|\mu_j, \mathbf{w}_\gamma, s_\gamma &\sim \text{Logit}\mathcal{N}\big(f_\gamma(\mu_j; \mathbf{w}_\gamma), s_\gamma\big) \\
\mu_j|\mathbf{x}_j, \mathbf{w}_\mu, s_\mu &\sim \text{Logit}\mathcal{N}\big(f_\mu(\mathbf{x}_j; \mathbf{w}_\mu), s_\mu\big) \\
\theta_{ij}|\mu_j, \gamma_j &\sim \text{Beta}(\mu_j, \gamma_j) \\
Y_{ij}|\theta_{ij}, n_{ij} &\sim \text{Binomial}(n_{ij}, \theta_{ij}) \\
f_\mu(\mathbf{x}_j; \mathbf{w}_\mu) &= \mathbf{w}_\mu^\top \mathbf{x}_j \\
f_\gamma(\mu_j; \mathbf{w}_\gamma) &= w_{\gamma 1} + \sum_{l=2}^{L} w_{\gamma l} g_l(\mu_j)
\end{aligned}
$$

Figure S24: Probabilistic graphical representation of the scMET model.

VGAM package. Then, $\mathbf{m}_{w\mu}$ values were set as the coefficients of the following linear regression model: $\text{logit}(\hat{\mu}_j) = \mathbf{m}_{w\mu}^\top \mathbf{x}_j + \epsilon_j$. For all analyses we assumed the feature-specific covariates were $\mathbf{x}_j = (1, C_j)$, where C_j denotes the CpG density. The $\mathbf{m}_{w\gamma}$ values were set as the coefficients of the following basis function regression model:

$$\text{logit}(\hat{\gamma}_j) = m_{w\gamma 1} + \sum_{l=2}^{L} m_{w\gamma l} g_l(\mu_j) + \epsilon_j \tag{5}$$

where $g_l(\cdot)$ are radial basis functions as defined in (7). The total number of basis functions L was fixed to 4 for all analyses; a choice flexible enough to capture the mean-variance relationship present in the data analysed in this study.

S2.3 EFDR calibration

The posterior evidence thresholds α quantify the uncertainty associated with the differential test or HVF analysis and can be fixed *a priori*. Otherwise, we can choose optimal thresholds to control the expected false discover rate (EFDR, 8) given by,

$$\text{EFDR}(\alpha) = \frac{\sum_{j=1}^{J} \big(1 - \pi_j(\psi)\big) \mathbb{I}\big(\pi_j(\psi) > \alpha\big)}{\sum_{j=1}^{J} \mathbb{I}\big(\pi_j(\psi) > \alpha\big)}, \tag{6}$$

where $\mathbb{I}(s) = 1$ if s is true, otherwise 0, α denotes the posterior evidence threshold and ψ the posterior probability. Unless otherwise stated, for DM and DV analysis we set EFDR = 5% and for HVF analysis we set EFDR = 10%. As discussed in (9), the usability of this calibration rule relies on the existence of features under both the null and alternative hypothesis (i.e. with and without changes in methylation patterns). As a default, if EFDR calibration is not achieved, we set $\alpha = 0.9$.

S2.4 Miscellaneous

Logit. If $p \in (0, 1)$ is a probability, the logit or log-odds function is defined as the logarithm of the odds,

$$\text{logit}(p) = \log\left(\frac{p}{1-p}\right). \tag{7}$$

Log-odds ratio. The logarithm of the odds ratio (LOR) is equal to the difference between the logits of two probabilities, that is:

$$\mathrm{LOR}(p_A, p_B) = \log\left(\frac{p_A/(1-p_A)}{p_B/(1-p_B)}\right) = \mathrm{logit}(p_A) - \mathrm{logit}(p_B). \tag{8}$$

F1-measure. The F1-measure or F1-score is the harmonic mean of precision and recall:

$$\text{F1-measure} = 2 \cdot \frac{\text{precision} \cdot \text{recall}}{\text{precision} + \text{recall}}. \tag{9}$$

Cluster purity. Purity is a measure of the extent to which clusters contain a single class. Given some set of clusters M and some set of classes D, both partitioning N data points, purity can be defined as:

$$\frac{1}{N} \sum_{m \in M} \max_{d \in D} |m \cap D|. \tag{10}$$

S2.5 Applying scMET with a sliding windows approach on the mouse brain cortex dataset

For the mouse brain cortex dataset (J=3,069 cells), we used 20kb windows and a step size of 20kb, yielding a total of 131,700 features. After applying the same filtering procedure described in *Methods*, this led to 82,309 features to be used as input for scMET. scMET was applied independently to each chromosome in parallel. Fig. S23 shows that the mean-overdispersion trends are effectively identical across chromosomes (we show 4 representative examples, but similar trends are observed for other chromosomes). This supports the use of residual overdispersion estimates as a metric for genome-wide HVF selection.

Name	Decouple mean-variance	Overdispersion	Feature selection	Diff mean	Diff variability	DOI
DSS	No	Yes	No	Yes	No	10.1093/nar/gku154
RADMeth	No	Yes	No	Yes	No	10.1186/1471-2105-15-215
BSmooth	No	No	No	Yes	No	10.1186/gb-2012-13-10-r83
RnBeads2.0	No	No	No	Yes	Yes	10.1186/s13059-019-1664-9
edgeR	No	Yes	No	Yes	No	10.12688/f1000research.13196.2
DMRcate	No	No	No	Yes	No	10.1186/1756-8935-8-6
MethylKit	No	No	No	Yes	No	10.1186/gb-2012-13-10-r87
scMET	Yes	Yes	Yes	Yes	Yes	

Table S1: Comparison of representative computational methods for analysing DNA methylation datasets.

References

[1] Carpenter B, Gelman A, Hoffman MD, Lee D, Goodrich B, Betancourt M, et al. Stan: A probabilistic programming language. Journal of Statistical Software. 2017;76(1). Available from: https://www.jstatsoft.org/article/view/v076i01.

[2] Plummer M, Best N, Cowles K, Vines K. CODA: Convergence diagnosis and output analysis for MCMC. R news. 2006;6(1):7–11. Available from: https://www.r-project.org/doc/Rnews/Rnews{_}2006-1.pdf.

[3] Luo C, Keown CL, Kurihara L, Zhou J, He Y, Li J, et al. Single-cell methylomes identify neuronal subtypes and regulatory elements in mammalian cortex. Science. 2017;357(6351):600–604. Available from: http://www.ncbi.nlm.nih.gov/pubmed/28798132.

[4] Argelaguet R, Clark SJ, Mohammed H, Stapel LC, Krueger C, Kapourani CA, et al. Multi-omics profiling of mouse gastrulation at single-cell resolution. Nature. 2019;576(7787):487–491. Available from: https://pubmed.ncbi.nlm.nih.gov/31827285/.

[5] Feng H, Conneely KN, Wu H. A Bayesian hierarchical model to detect differentially methylated loci from single nucleotide resolution sequencing data. Nucleic Acids Research. 2014;42(8):e69. Available from: https://pubmed.ncbi.nlm.nih.gov/24561809/.

[6] Gelman A, Carlin JB, Stern HS, Dunson DB, Vehtari A, Rubin DB. Bayesian data analysis. CRC Press; 2013. Available from: https://doi.org/10.1201/b16018.

[7] Kapourani CA, Sanguinetti G. Higher order methylation features for clustering and prediction in epigenomic studies. Bioinformatics. 2016;32(17):i405–i412. Available from: http://www.ncbi.nlm.nih.gov/pubmed/27587656/.

[8] Newton MA, Noueiry A, Sarkar D, Ahlquist P. Detecting differential gene expression with a semiparametric hierarchical mixture method. Biostatistics. 2004;5(2):155–176. Available from: http://www.ncbi.nlm.nih.gov/pubmed/15054023/.

[9] Vallejos CA, Richardson S, Marioni JC. Beyond comparisons of means: understanding changes in gene expression at the single-cell level. Genome Biology. 2016;17(70):1–14. Available from: http://www.ncbi.nlm.nih.gov/pubmed/27083558/.
