# Supplementary material for: scMET: Bayesian modeling of DNA methylation heterogeneity at single-cell resolution
Source: Genome Biol. 2021 Apr 20;22:114. doi: 10.1186/s13059-021-02329-8 (PMC8056718; doi:10.1186/s13059-021-02329-8)
Supplement: Supplementary file 6 — Additional file 6 Review history. [file 13059_2021_2329_MOESM6_ESM.docx]

Review History

**First round of review**

**Reviewer 1**

**Were you able to assess all statistics in the manuscript, including the appropriateness of statistical tests used?**

Yes: The statistics and models in the manuscript are appropriate for the research topic and the scope of the study.

**Were you able to directly test the methods?**

Yes

**Comments to author:**

The manuscript described a bayesian hierarchical model, scMET, to characterize single cell DNAm on genome features, trying to disentangle genuine epigenetic variability form technical biases. I believe this is a useful tool can be useful but the manuscript could be improved and draw more interests if the following points were addressed:

1. One of the most important but still widely open question of single cell DNAm data is how we could determine cell type specific cis regulatory elements from the data. Thus it is more interesting to determine genome regions with differential methylation level/variability in a de novo way compared to using pre-annotated genome regions as the manuscript reported. The manuscript did mention sliding windows can be used, but it would be really helpful if thorough testing using sliding window as features could've been reported such as running time for genomewide test, how window size affects performance, etc.

2. What are the length distribution of the pre-annotated features? Is there any performance biased with the feature length?

3. scMET models both methylation level and variability of a single genome feature using a single parameter (theta and gamma, respectively). I understand the rationale and the need of simplification when features are above a certain length. However, for genome features like regions with histone modification peaks, the methylation heterogeneity of CpG sites within one region can be profound. As the method attempts to address the sparsity and variability of sc data, it would be helpful if the authors could comment on what criteria (read length, coverage, feature length etc) make valid input features and the limitation of the method if any.

4. a question related to 2,3. In addition to how cell number affects methylation level/variability estimation (Fig S5), it would be helpful to see the performance distributions against feature length for different cell numbers

5. For the use case of highly variable feature (HVF) selection, it will be helpful to benchmark with less sophisticated also dispersion-based methods like those used widely in RNA analysis, for example Zheng17 or Satij15, which was adopted in sc-DNAm data analysis Liu&Zhou20.

Zheng et al. (2017), Massively parallel digital transcriptional profiling of single cells, Nature Communications.

Satija et al. (2015), Spatial reconstruction of single-cell gene expression data, Nature Biotechnology.

Liu&Zhou et al. (2020), DNA Methylation Atlas of the Mouse Brain at Single-Cell Resolution, biorxiv

6. for the analysis of both DNAm and RNA, the variability of the two modality did not show a clear relationship. In addition to variability, is there a relation between promoter DNAm level and RNA expression level in this dataset? I also wonder how it will look like if DNAm of fthe irst introns or exons were used from this same data set (Brenet11, Anastasiadi18)?

Brenet et al. (2011) DNA Methylation of the First Exon Is Tightly Linked to Transcriptional Silencing, Plos ONE

Anastasiadi et al. (2018) Consistent inverse correlation between DNA methylation of the first intron and gene expression across tissues and species, BMC Epigenetics & Chromatin

**Reviewer 2**

**Were you able to assess all statistics in the manuscript, including the appropriateness of statistical tests used?**

No

**Were you able to directly test the methods?**

Yes

**Comments to author:**

Kapourani, Argelaguet, et al. present a paper describing scMET, a tool for the statistical analysis of single-cell DNA methylation data that promises to overcome problems inherent to the technology (i.e., extreme data sparsity) by sharing information across cells, which should enable robust analysis of differential DNA methylation patterns as well a differential variability. To date, papers reporting single-cell DNA methylation data are still relatively rare (compared to single-cell transcriptome data), which is due to the relative inaccessibility of the technology to most labs - which is due to both the technology itself but also due to the difficulty of performing meaningful analysis of the data. I therefore believe development of improved statistical methods, such as the one presented here, could be of great value to the community.

My overall impression is very positive. The method presented seems sound and useful. I particularly like the promise to analyse differential variability without confounding impact of differences in means (albeit I have some doubt about how well it works - see below), and the ability to incorporate other confounders (CpG density, motifs, etc.) in the model. and the paper is for the most part well written and understandable. The figures are also of a good quality. However, I am not satisfied with the comparison to the state-of-the-art and have a few suggestions for clarifications. I think the authors should be able to address all points fairly quickly.

Major concerns:

1. Given that this is primarily a methods paper, my main concern pertains to the lack of comparison to existing, related methods. The authors present a number of use cases on simulated and real data, including example results on specific genes - which is all very nice to make a point that scMET may produce biologically meaningful results. However, from what is presented in the paper it is impossible to grasp what existing alternative tools are out there and what I actually specifically gained from using scMET.

Trying to provide constructive criticism, I have identified at least three points that should be addressed to put scMET better into context:

(Dis-)advantages of using sliding windows and genomic features are insufficiently discussed. Relatedly, a rather popular approach seems to be to define data-driven features with consistent DNA methylation patterns in the given dataset. This approach has also already been used for scDNAm data, e.g. using BSSmooth (https://doi.org/10.1186/gb-2012-13-10-r83, https://doi.org/10.1016/j.stemcr.2018.07.003). This approach (and why it scMETs approach is superior, if so) should be discussed.

Feature selection: A comparison to randomly selected features would help to interpret the goodness of the features selected by scMET (Fig. 2b). Also, in the context of Fig. 2, would it be appropriate to compare (at least in words) to MOFA (of which one of the developers is an author on this manuscript), Epiclomal (https://doi.org/10.1371/journal.pcbi.1008270), or the recent pre-print by the Tan lab (https://doi.org/10.1101/2020.06.05.137000). If not, why?

Most important, in my opinion, is a comparison to existing tools for differential methylation analysis from BS-seq data, even if not developed specifically for single-cell data. In terms of performance and practical aspects (runtime, scope, platform, …). The comparison to Fisher's test is the very baseline and not sufficient. This is critical for the reader to appreciate the contribution made with scMET and to make an informed decision about the tools to use. Without any claims for comprehensiveness, tools to be considered may include (non-comprehensive list):

Dmrseq https://doi.org/10.1101/183210

DSS https://doi.org/10.1007/s40484-019-0183-8

RnBeads https://doi.org/10.1186/s13059-019-1664-9

edgeR https://doi.org/10.12688/f1000research.13196.2

DMRcate https://doi.org/10.1186/1756-8935-8-6

2. While I appreciate the effort to "introduce[s] residual overdispersion estimates as a measure of DNAm variability that is not confounded by DIFFERENCES in mean methylation" (p. 2, l. 31), I wonder whether this claim actually holds. Looking at Fig. 4, it seems to me that the shown features with high residual DNA methylation overdispersion, are also marked by differences in mean DNA methylation level. This is particularly striking for Dppa5a and Spp1, but likely also for the other two (Fig. 3b). Depending on whether my interpretation is correct, this might be either (a) major concern with the appropriateness of the model requiring further improvement, convincing data / analysis, or removing the "critical" claim from the paper, or (b) a minor concern that could be resolved my clarifying the misunderstanding (e.g., if the authors just mean to say "not confounded by mean DNA methylation levels" as opposed to "differences in mean methylation").

Minor concerns:

- The term "residual overdispersion" is quite important for the paper (first mention, l. 31), yet may not be common knowledge for many readers. Further (high-level) explanation would be beneficial. Perhaps, the first paragraph of the Methods should be included in the main text (introduction?).

- We are told scMET uses a Beta Binomial model, but it is not clear whether this model was chosen among other options for a particular reason or under some specific assumptions about the data (p. 3). Again, the meaning of this term should be briefly explained and a justification given (is it common knowledge [reference??]; is it empirical? Based on some prior model selection on the distributions of real datasets?)

- In defining the parameters of the model, the authors need to be very careful about the wording as this can be easily confusing readers not intimate to these data. In my experience, even most "genomics experts" come in their thinking from a perspective of transcriptomics, where it's all about counting reads, and fail to immediately grasp the essentially binary nature of DNA methylation and the fact that there's a limited number of DNA fragments that could have ever been measured in a single cell. "For each cell i and feature j, the input for scMET is the number of CpG sites that are observed to be methylated (Yij ) and the total number of sites for which methylation status was recorded (nij )" - what the authors mean by "observed to be methylated" and "total […] recorded" is the number of CpG sites that were covered by a read (as opposed to the many NAs that were not covered at all). Those CpGs that were covered can either be methylated or not. I'd urge the authors to carefully consider / optimize their explanations here. Perhaps some of Figure 1b could be better used to illustrate this.

- p.2, l. 24: "To overcome sparsity, scMET aggregates the input data within regions (hereafter also referred to as genomic features): either by combining CpG information in a sliding window approach or using pre-annotated contexts". Details should be given about the sliding window approach (which window sizes? Any restrictions? Does it matter?), and the analysis in Fig. 3 and 4 be performed also with these sliding windows (in addition to the pre-defined features).

- The authors state that scMET is being prepared for submission to Bioconductor. That would be much appreciated. Can the dependency on v8 be dropped? I don't understand why a Javascript engine would be required for scMET and the dependency makes installation more troublesome (because most users won't have the system-level v8 dependency pre-installed).

**Authors Response**

**Point-by-point responses to the reviewers’ comments:**

Reviewer 1

The manuscript described a Bayesian hierarchical model, scMET, to characterize single cell DNAm on genome features, trying to disentangle genuine epigenetic variability form technical biases. I believe this is a useful tool can be useful but the manuscript could be improved and draw more interests if the following points were addressed.

*We thank the reviewer for this supportive statement and insightful comments.*

1. One of the most important but still widely open question of single cell DNAm data is how we could determine cell type specific cis regulatory elements from the data. Thus it is more interesting to determine genome regions with differential methylation level/variability in a de novo way compared to using pre-annotated genome regions as the manuscript reported. The manuscript did mention sliding windows can be used, but it would be really helpful if thorough testing using sliding window as features could’ve been reported such as running time for genome-wide test, how window size af¬fects performance, etc.

*Following the reviewer’s suggestion, we have revised the manuscript to demonstrate the use of sliding windows as input to scMET. We introduce a modified analysis pipeline that enables a computationally feasible approach in this context and apply it to the mouse brain cortex dataset [1]. These additions have substantially strengthened the manuscript and are described in detail below.*

*A scalable strategy to use scMET with genome-wide sliding windows as input*

*By pooling information across cells and features, scMET leads to more robust variability estimates than simpler methods which analyse each feature independently. We addressed the increased compu-tational complexity by adopting a variational Bayes algorithm which scales linearly with the number of features, enabling the analysis of large-scale data sets (Figure S2). But additional challenges arise due to the increased dimensionality introduced by using the sliding windows approach with a genome-wide scale. For example, for the mouse genome, using sliding windows of 20kb with a step size of 20kb yields approximately 130,000 features. This may preclude the use of scMET within a practical timeline for large scale applications. In the spirit of divide-and-conquer schemes [2], we bypass this problem via a parallelisation strategy in which we apply scMET separately to each chro¬mosome. Feature-specific estimates obtained for each chromosome are then combined post-hoc when performing highly variable feature (HVF) selection and differential analyses.*

*We suggest careful consideration when selecting the input feature set whilst using the sliding window*

*approach. First, it is critical to apply a quality control step to remove windows with very low coverage (number of CpGs whose methylation status was observed), for which inference is less reliable. Second, the choice of window size can restrict the type of regions to be included in the analysis. For example, regulatory elements such as enhancers tend to be relatively small, spanning from a hundred to a few thousand base pairs [3]. Such regions would be excluded when using large window sizes. In contrast, small windows may fail to have sufficient coverage. Data-driven approaches could be used to identify an optimal window size (see suggestion by Reviewer 2). For example, [4] identified that 2kb windows are sufficient to capture regions with high CpG-to-CpG concordance. An alternative solution would be to iteratively refine the window size whilst performing downstream analyses. This was implemented in metilene [5] in the context of differential mean methylation testing for bulk DNAm sequencing data. Implementation of such iterative strategy for differential variability testing and HVF selection whilst taking into account the sparsity of single cell DNAm data is a very interesting direction for future research, but we believe that this is beyond the scope of the current study. We have added a section in Methods to describe the approach introduced above.*

*Applying scMET with a sliding windows approach on the mouse brain cortex dataset For these data (J=3,069 cells), we used 20kb windows and a step size of 20kb, yielding a total of 131,700 features. After applying the same filtering procedure described in Methods, this led to 82,308 features to be used as input for scMET. scMET was applied independently to each chromosome in parallel. Figure R1 shows that the mean-overdispersion trends are effectively identical across chromo¬somes (we show 4 representative examples, but similar trends are observed for other chromosomes). This supports the use of residual overdispersion estimates as a metric for genome-wide HVF selection.*

*As shown in Figures R2a and R2e, HVFs selected amongst 20kb sliding windows led to similar results as when using pre-annotated H3K27ac and H3K4me1 features as input (Figure 2b). This suggests that the selected windows can distinguish most of the different cellular sub-populations. However, we observe slightly lower ARI values, suggesting that pre-annotated features can better recapitulate the underlying population structure. Notably, consistent with our results when using ChIP-seq peaks, the HVF selection by scMET better captures the underlying cellular sub-populations than those selected using the variance from a Gaussian model, which is still the most commonly used strategy [6, 7, 8].*

*Among the windows with the largest residual overdispersion, we identified putative regulatory el-ements that discriminate cell types and that are located near neuronal marker genes (Figure R2b and R2c). As an example, chr1:56940001-56960001 (Figure R2d, top) is located within the intronic region of Satb2, a marker of excitatory neurons [1]. As expected, this region is unmethylated in most excitatory neurons and highly methylated in inhibitory neurons. In contrast, chr1:68780001-68800001 (Figure R2d, bottom) is located within the intronic region of Erbb4, a marker of inhibitory neurons [1]. This region is highly methylated in all excitatory neurons and only partially methylated in inhibitory neurons.*

*Finally, Figure R3 shows the scalability of scMET on the mouse brain cortex dataset across different sliding window settings. When using 10kb or 20kb windows across the whole dataset (-3,000 cells), the running time of scMET is around one day per chromosome. In contrast, the running time when focusing on the inhibitory cell population (-400 cells) is around 4 hours per chromosome. Hence, users may consider the use of pre-annotated regions (as in our original analysis) when characterising cell sub-populations within a large scale data set, leaving the use of a sliding windows approach to more targeted analysis within specific sub-populations of interest. We note that the current version of scMET is based in RStan. We defer for future work a more efficient bespoke implementation of scMET that will facilitate genome-wide analysis on large scale single cell DNAm studies.*

*Figure R1. Mean-overdispersion relationship for the mouse brain cortex dataset, across four different chromosomes. Left sub-panels show posterior medians for feature-specific mean (µj, x-axis) and overdispersion (γj, y-axis) param¬eters. Black line represents the estimated regression trend from the GLM component of scMET. Right sub-panels show posterior medians for feature-specific methylation parameters (µj, x-axis) versus residual overdispersion pa¬rameters (cj, y-axis). Each data point corresponds to a different feature. The color code within the scatter-plots is used to represent areas with high (green and yellow) and low (blue) concentration of features. For simplicity we show 4 representative examples, but similar trends are observed for the remaining chromosomes*

*Figure R2. Identifying genome-wide HVFs from 20kb sliding-windows in the mouse brain cortex dataset. (a) Clustering performance in terms of adjusted Rand index (ARI) for varying number of HVFs. HVF selection was based on scMET’s residual overdispersion parameters j (yellow), Gaussian variance (grey) and normalised dispersion (NormDisp, cyan). A finite grid of HVFs was used for ARI evaluation and non-parametric regression was used to obtain a smoothed interpolation across all values. (b) Mean methylation (µj, x-axis) is plotted against overdispersion estimates (γj, y-axis). Red points correspond to features being called as HVF by scMET. (c) Histogram showing enrichment distribution under the null model. The null model is based on randomly labelling features as HVFs and then counting how many of those are overlapping with neuron marker genes identified by [1], see Supplementary Table S1. To obtain a null distribution we repeated this process 1,000 times. The dashed vertical yellow line, shows the enrichment of HVFs using the scMET model. (d) Examples of windows with large residual overdispersion which overlap with Satb2 (top) a marker gene for excitatory neurons, and Erbb4 (bottom) a marker gene of inhibitory neurons. Left sub-panels stratify cells by the broad neuronal class. Right sub-panels group cells by the neuronal subclass. (e) UMAP representation for varying number of HVFs when applied to 20kb sliding windows on the mouse brain cortex dataset. scMET was run independently per chromosome. Top HVF features were subsequently identified on the genome-wide dataset based on residual overdispersion parameters j (see Methods).*

*Figure R3. scMET scalability analysis on the mouse brain cortex dataset for different sliding window lengths and total number of cells. scMET was run independently (in parallel) for each chromosome for a maximum of 20,000 iterations. Boxplots present the distribution of running times for scMET (in hours) across chromosomes.*

2. What are the length distribution of the pre-annotated features? Is there any performance biased with the feature length?

*Figure R4 displays the length distribution across features for the pre-annotated distal H3K27ac and H3K4me1 genomic contexts. As seen in Figures R5 and R6, we do not observe feature length to introduce biases in parameter estimates obtained by scMET, nor does feature length appear to affect the results of our HVF selection approach or differential methylation analyses.*

*We should note that scMET is a versatile tool that can incorporate any feature-specific covariates that explain differences in mean methylation levels across features. Therefore, in cases where the effect of feature length is of interest, users can include feature length as an additional covariate.*

*2. What are the length distribution of the pre-annotated features? Is there any performance biased with the feature length?*

*Figure R4 displays the length distribution across features for the pre-annotated distal H3K27ac and H3K4me1 genomic contexts. As seen in Figures R5 and R6, we do not observe feature length to introduce biases in parameter estimates obtained by scMET, nor does feature length appear to affect the results of our HVF selection approach or differential methylation analyses.*

*We should note that scMET is a versatile tool that can incorporate any feature-specific covariates that explain differences in mean methylation levels across features. Therefore, in cases where the effect of feature length is of interest, users can include feature length as an additional covariate.*

3. scMET models both methylation level and variability of a single genome feature using a single pa-rameter (theta and gamma, respectively). I understand the rationale and the need of simplification when features are above a certain length. However, for genome features like regions with histone modification peaks, the methylation heterogeneity of CpG sites within one region can be profound. As the method attempts to address the sparsity and variability of sc data, it would be helpful if the authors could comment on what criteria (read length, coverage, feature length etc) make valid input features and the limitation of the method if any.

*We agree that DNAm heterogeneity across CpG sites within a region might arise and that it can be profound for regions with histone modification peaks. scMET focuses on the quantification of cell-to-cell heterogeneity at the region level, but complementary approaches such as Melissa [9] and BPRMeth [10] could be used to characterise CpG-to-CpG heterogeneity within each region. However, CpG-level data is substantially more sparse and has dimensions that are considerably larger than datasets in which CpGs are aggregated per region. In such cases, scMET could be used to identify specific regions of interest (e.g. high residual overdispersion) to be used as the input for more targeted analyses of CpG-to-CpG heterogeneity.*

*In terms of the input data requirements for scMET, the most important step is to perform quality control to remove features with very low CpG coverage (the number of CpG sites for which methy-lation status was captured within a feature). For example, our analyses only included features with sufficient coverage (at least 3 CpGs) in at least 15 cells. Note that differences in coverage may capture variability in feature length (more CpGs in longer regions) and that read length can affect coverage. Hence, we focus on demonstrating the performance of scMET for features with varying coverage.*

*First, we use synthetic data (see Methods), grouping features according to the average coverage CpGs across all cells (Figure R7). For each feature, we computed the posterior standard deviation for the residual overdispersion parameter j. Large values (e.g. above 0.5) indicate higher uncertainty in our estimates, and may affect the robustness of downstream analyses. Based on this analysis, we suggest to exclude regions that have less than 3 CpGs covered (on average across all cells) for relatively large sample sizes ( 100 cells), with a higher threshold of 5 CpGs for small datasets ( 50 cells).*

4. A question related to 2,3. In addition to how cell number affects methylation level/variability esti¬mation (Fig S5), it would be helpful to see the performance distributions against feature length for different cell numbers.

*As discussed above, coverage is the key data property that is relevant for the performance of scMET. Variations in coverage can be used as a proxy to assess the effect of feature length, as larger regions will tend to have higher number of CpGs. Hence, the comparison presented in Figure S5 (original submission) can be used in this context. As expected, we can conclude that estimation performance is higher for longer features (see CpG rich in original Figure S5). Importantly, when comparing to beta-binomial MLE estimates, we observe that scMET provides more stable overdispersion estimates, particularly for small sample sizes and shorter regions (CpG poor in original Figure S5). Among others, this is essential for studies focusing on methylation heterogeneity of histone modification peaks (as per reviewer’s item 3), where we expect scMET to be used extensively. To mimic shorter regions (e.g. peaks of regulatory elements), we now include a new simulation experiment in which features have an average coverage of 5 CpGs (see top panels in Figure R8, which now replaces original Figure S5 (Figure S6 in the revised manuscript)). In this context, the beta-binomial MLE estimates resulted in particularly poor performance. This showcases the advantages of using scMET, particularly in scenarios with small sample sizes (see also Figure S7 on down-sampling experiment) and shorter features, for which coverage is low.*

*Figure R8. Shrinkage effect on posterior estimates for feature-specific overdispersion parameters γj. (a, b, c) Overdispersion estimates per feature for varying number of cells using the beta-binomial MLE (grey) and scMET (yellow) models. For scMET each point represents the posterior median for the associated parameter. Vertical arrows denote the shrinkage that is introduced by scMET with respect to MLE estimates. The dashed line corresponds to perfect agreement between true (x-axis) and estimated (y-axis) overdispersion. (a) CpG poor regions, (b) CpG moderate regions, and (c) CpG rich regions. (c) For the synthetic data in (a), (b) and (c), we use the absolute log-odds ratio (LOR) difference between true and estimated values as a measure of estimation performance for varying number of cells. The smaller the LOR value the better the estimation performance. Each data point represents a feature.*

5. For the use case of highly variable feature (HVF) selection, it will be helpful to benchmark with less sophisticated also dispersion-based methods like those used widely in RNA analysis, for example Zheng17 or Satija15, which was adopted in sc-DNAm data analysis Liu&Zhou20.

*Following the Reviewer’s suggestion, we implemented the normalised dispersion approach widely used in scRNA-seq (here termed NormDisp). We did this by adopting the code that was used by Zheng17 (github.com/10XGenomics/single-cell-3prime-paper/blob/master/pbmc68k analysis/util.R). In addition, as per Reviewer’s 2 suggestion, we include an additional comparison with respect to a Random approach, in which HVFs are selected randomly within the input set. HVFs selected by scMET achieve the highest clustering performance across the three selected genomic contexts for the mouse frontal cortex dataset (Figure R9). Figure R9 now replaces Figure 2b in the main text.*

*To gain a better understanding about the HVF selection implemented by each method, an additional comparison is provided in Figure R10a. Similar to scMET, the NormDisp approach selects HVFs that are not that confounded by the mean-overdispersion relationship. However, NormDisp HVFs exhibit poorer clustering performance. This potentially occurs due to the normalised dispersion point estimates being more unstable. For instance, when analysing the discrepancies among the top 200 HVFs selected by NormDisp and scMET, we observe that the top three HVFs called by NormDisp (but not called by scMET) appear to be driven by outliers (Figure R10b). In contrast, top HVFs called by scMET (but not NormDisp) show a bimodal distribution and are able to better recapitulate the underlying population structure. This discussion has been added to the revised manuscript.*

*Figure R9. Clustering performance in terms of adjusted Rand index (ARI), for varying number of HVFs. HVF selection was based on scMET’s residual overdispersion parameters j (yellow), binomial variance (grey), Gaussian variance (cyan), normalised dispersion (NormDisp, red) and random selection (blue). A finite grid of HVFs was used for ARI evaluation and non-parametric regression was used to obtain a smoothed interpolation across all values.*

*Figure R10. (a) Mean-overdispersion relationship for the distal H3K27ac genomic context, as estimated by scMET. Red points correspond to top 200 HVFs being called by the corresponding method given in the title of each subplot. (b) Top three HVFs, based on highest normalised dispersion value, as computed by the NormDisp method. These features were not called as HVF by scMET. (c) Top three HVFs (that were not called by NormDisp) based on scMET’s residual overdispersion.*

6. For the analysis of both DNAm and RNA, the variability of the two modality did not show a clear relationship. In addition to variability, is there a relation between promoter DNAm level and RNA expression level in this dataset?

*First, we explored the relationship between promoter DNAm and RNA expression mean levels. As shown in Figure R11, when averaging across all cells, these two modalities show a statistically significant negative correlation (Pearson’s correlation R = -0.4). This correlation is mostly driven by two groups of features: those with low mean methylation and high mean expression (upper left), and a smaller subset of promoters with high mean methylation and low gene expression (bottom right). In addition, we quantified a Pearson’s correlation coefficient between promoter DNAm and RNA expression for each individual cell. This yielded a negative correlation for all cells (Figure R12), in line with previous studies that correlated promoter DNAm and RNA expression in individual embryonic stem cells [6, 7].*

*Figure R11. Scatter plot displays posterior median estimates for mean DNAm parameters µj in gene promoters (x-axis) versus RNA mean expression of the corresponding genes (y-axis). Each data point corresponds to a different feature. The color code within the scatter-plots is used to represent areas with high (green and yellow) and low (blue) concentration of features.*

*Figure R12. Histogram of Pearson correlation coefficient R between RNA expression and promoter DNAm, quan¬tified for each cell independently.*

*Note that the type of association analysis shown in Figure R12 is performed per cell (or per sample, in the case of bulk measurements). The availability of single-cell multi-modal data enables us to perform associations per loci (across cells), which is the most appropriate analysis for the identifica-tion of putative regulatory elements that co-vary with RNA expression changes. When calculating a correlation coefficient per loci we observe that most genes do not display a significant correlation between DNAm and RNA expression (see [6, 7]), consistent with the scatter plot of RNA expression and promoter DNAm residual overdispersion estimates shown in Figure 4a.*

7. I also wonder how it will look like if DNAm of the first introns or exons were used from this same data set (Brenet11, Anastasiadi18)?

*As suggested by the reviewer, we explored the relationship between residual over-dispersion estimated derived for RNA expression and DNAm using the first exon and intron. Similar to the promoter DNAm analysis shown in Figure 4a, there is no clear association between the residual overdispersion estimates on both genomic contexts (Figures R13 and R14).*

*Figure R13. Scatter plot displays posterior median estimates for residual DNAm overdispersion parameters j in first exons (x-axis) versus RNA residual overdispersion of the corresponding genes (y-axis). The list of genes is the same as in Figure 4 in main text.*

*Figure R14. Scatter plot displays posterior median estimates for residual DNAm overdispersion parameters j in first introns (x-axis) versus RNA residual overdispersion of the corresponding genes (y-axis). The list of genes is the same as in Figure 4 in main text.*

Reviewer 2

Kapourani, Argelaguet, et al. present a paper describing scMET, a tool for the statistical analysis of single-cell DNA methylation data that promises to overcome problems inherent to the technology (i.e., extreme data sparsity) by sharing information across cells, which should enable robust analysis of differential DNA methylation patterns as well a differential variability. To date, papers reporting single-cell DNA methylation data are still relatively rare (compared to single-cell transcriptome data), which is due to the relative inaccessibility of the technology to most labs - which is due to both the technology itself but also due to the difficulty of performing meaningful analysis of the data. I therefore believe development of improved statistical methods, such as the one presented here, could be of great value to the community.

My overall impression is very positive. The method presented seems sound and useful. I particularly like the promise to analyse differential variability without confounding impact of differences in means (albeit I have some doubt about how well it works - see below), and the ability to incorporate other confounders (CpG density, motifs, etc.) in the model. and the paper is for the most part well written and understandable. The figures are also of a good quality. However, I am not satisfied with the comparison to the state-of-the-art and have a few suggestions for clarifications. I think the authors should be able to address all points fairly quickly.

*We thank the reviewer for highlighting the importance of our work and the constructive feedback.*

1. Given that this is primarily a methods paper, my main concern pertains to the lack of comparison to existing, related methods. The authors present a number of use cases on simulated and real data, including example results on specific genes - which is all very nice to make a point that scMET may produce biologically meaningful results. However, from what is presented in the paper it is impossible to grasp what existing alternative tools are out there and what I actually specifically gained from using scMET. Trying to provide constructive criticism, I have identified at least three points that should be addressed to put scMET better into context:

(a) (Dis-)advantages of using sliding windows and genomic features are insufficiently discussed. Re-latedly, a rather popular approach seems to be to define data-driven features with consistent DNA methylation patterns in the given dataset. This approach has also already been used for scDNAm data, e.g. using BSmooth (https://doi.org/10.1016/j.stemcr.2018.07.003). This approach (and why it scMETs approach is superior, if so) should be discussed.

*As part of the revised manuscript, we illustrate the use of sliding windows for the mouse brain cortex data. This is described in detail in our response to Reviewer 1, item 1. In brief, clustering performance based on HVF selected using 20 kb sliding windows as input is similar (albeit slightly lower) that what we obtained for the pre-annotated regions. We also proposed a divide-and-conquer strategy (chromosome-by-chromosome) to address the substantially larger number of input features introduced by the sliding windows approach (≈ 130,000 features for the mouse genome when using 20kb windows and a step size of 20kb).*

*The use of the sliding windows strategy requires the window size to be set a priori. Large regions reduce the granularity of the data and could mask small regulatory elements (e.g. enhancers spanning from hundreds to a few thousand base pairs [3]). In contrast, small windows are likely to lack sufficient coverage in single cell DNAm sequencing data. As mentioned by the reviewer, a data-driven approach could help to identify an optimal window size. In particular, the suggested reference [4] shows that 2kb windows can define regions with high CpG-to-CpG concordance. After appropriate quality control (to remove regions with low coverage), 20kb windows (or any other user selected window size) could be used as the input for scMET. An alternative solution would be to iteratively refine the window size whilst performing downstream analyses. This approach is used by metilene [5] in the context of differential mean DNAm testing for bulk-level data. Implementation of such iterative strategy for differential variability testing and HVF selection whilst taking into account the sparsity of single cell DNAm data is an interesting direction for future research, but we believe that this is beyond the scope of the current study.*

*In [4], PDClust is introduced as a clustering tool for single cell DNAm data. PDClust uses PD values as the distance metric between pairs of cells. These are defined as the average dissimilarity between CpGs with common coverage (i.e. CpGs with no coverage in either cell are excluded from this calculation). This may be problematic for highly sparse data (e.g. shallow sequencing of large numbers of cells), for which only a small number of CpGs are likely to contribute to the calculation of PD values. Moreover, the choice between a genome-wide analysis and a targeted analysis (in which input CpGs are restricted to pre-selected regions of interest) can affect the ability to separate known cell types (Figure 3C-F in [4]). Hence, scMET and PDClust could be used as complementary tools, with HVFs identified by scMET used as the input for PDClust.*

*Finally, we note that data-driven differentially methylated regions (DMRs) defined by BSmooth [11] are obtained by performing differential mean methylation analysis at the CpG level. Sub¬sequently, DMRs are called by merging differentially methylated CpGs that are located close in genomic space (within 500bp of distance in the reference provided by the reviewer). This post-hoc merging strategy enables the definition of data-driven features which could, in princi¬ple, be used as the input for scMET’s differential methylation analyses. Having said that, such approach would be challenging for single-cell methylation data, as it requires a relatively high coverage, which is only available in bulk assays (and even in bulk assays some methods such as BSmooth require the use of data smoothing techniques). In addition, DMRs are only driven by mean differences between known groups of cells. As such, these would fail to capture differ¬entially variable regions and cannot be used for unsupervised exploration of single-cell DNAm data with unknown sub-population structure.*

(b)	Feature selection: A comparison to randomly selected features would help to interpret the good¬ness of the features selected by scMET (Fig. 2b).

*As suggested by the reviewer, we have updated Figure 2b to include a Random approach, in which HVFs are selected randomly within the input set. This comparison is also provided in Figure R9 within this report (see also the response to reviewer 1, item 5). In all three genomic contexts, we observe that scMET outperforms the random feature selection approach.*

(c)	Also, in the context of Fig. 2, would it be appropriate to compare (at least in words) to MOFA (of which one of the developers is an author on this manuscript), Epiclomal (https://doi.org/10.1371/journal.pcbi.1008270), or the recent pre-print by the Tan lab (https://doi.org/10.1101/2020.06.05.137000). If not, why?

*We apologise for the confusion. MOFA is a dimensionality reduction method based on Bayesian Group Factor Analysis that provides different (but complementary) functionalities than scMET. In particular, we propose to use scMET for the feature selection step, prior to dimensionality reduction with MOFA. In fact, a principled feature selection step leads to more informative latent representations and clustering results (Figure 2b). Other dimensionality reduction tools such as Principal Component Analysis (PCA) or Non-negative Matrix Factorisation (NMF) could be used instead. However, the advantage of MOFA is that it naturally handles missing methylation values (features with no CpG sites covered on certain cells, i.e. the censored values described in the Methods) by ignoring the corresponding terms in the likelihood function. This does not represent a major problem for bulk DNAm assays, but it is a very important and some¬times underappreciated challenge in single-cell DNAm analyses. Instead, most implementations of conventional dimensionality reduction methods (e.g. PCA and NMF) require imputation of missing values, which can be dangerous for very sparse datasets.*

*A similar situation arises for the other methods mentioned by the reviewer. Epiclomal is a proba-bilistic clustering and imputation method using a hierarchical mixture model. Instead, MAPLE (the method by the Tan’s lab) is aimed at detecting correlations between RNA expression and DNA methylation using a supervised learning model. This enables cell type assignments by transferring labels from scRNA-seq data sets to single-cell methylation data. In both cases, scMET can be used to select a relevant set of input features defined by identifying regions with high DNAm variability that can better characterise the underlying population structure.*

*We hope that this discussion clarifies the reviewer’s concern. In the Discussion section of the revised manuscript, we comment on these methods and how scMET could be incorporated as a complementary approach in the analysis pipeline.*

(d) Most important, in my opinion, is a comparison to existing tools for differential methylation analysis from BS-seq data, even if not developed specifically for single-cell data. In terms of performance and practical aspects (runtime, scope, platform, ...). The comparison to Fisher’s test is the very baseline and not sufficient. This is critical for the reader to appreciate the con¬tribution made with scMET and to make an informed decision about the tools to use. Without any claims for comprehensiveness, tools to be considered may include (non-comprehensive list): Dmrseq; DSS; RnBeads; edgeR; DMRcate.

*We agree with the reviewer: a comparison with existing DNA methylation analysis tools (primar¬ily developed for bulk assays) could provide additional insights when evaluating the performance of scMET. We note, however, that is important to emphasise fundamental differences with re¬spect to the functionality provided by scMET. These are summarised in Table R1 (see also Supplementary Table S1) and the updated Methods. In particular, the approaches suggested by the reviewer focus on differences in mean methylation. Existing methods are often based on some version of a t-test on the continuous mean methylation rates (DMRcate, BSmooth); a Fisher test on the methylation counts (e.g. MethylKit, RnBeads); negative binomial models for methylation counts (often adapted from the bulk RNA sequencing literature, e.g. edgeR); or on beta-binomial models (e.g. DSS). As seen in Figure S5, both scMET and beta-binomial*

*MLE estimates for mean methylation parameters are comparable. As it can be expected, our comparison with respect to DSS also led to similar results (Figure R15). When comparing with respect to the Fisher’s exact test, we did not observe substantial differences in performance either: scMET was better in terms of the F1-measure and type I error control, but led to more conservative results (Supplementary Fig. S8 and S9). Therefore, we do not aim to claim overall superiority of scMET for differential mean methylation testing. If a user is only interested in differential mean methylation, existing approaches such as DSS could be used. Instead, the main advantage of scMET is the ability to robustly perform differential variability testing (for which scMET is the first approach tailored to single cell DNAm data) and HVF selection (see extensive comparison in Figure 2b, Supplementary Figure S14b). For clarity, this discussion is now included as part of the Results section.*

*Figure R15. Comparing differential methylation estimates between scMET and DSS. (left) Comparing differential mean levels (point estimates) between DSS (x-axis) and scMET (y-axis). Shown is the y=x line. (right) Comparing log p-values inferred by DSS (x-axis) with differential tail probabilities inferred by scMET (y-axis)*

2. While I appreciate the effort to ”introduce[s] residual overdispersion estimates as a measure of DNAm variability that is not confounded by DIFFERENCES in mean methylation” (p. 2, l. 31), I wonder whether this claim actually holds. Looking at Fig. 4, it seems to me that the shown features with high residual DNA methylation overdispersion, are also marked by differences in mean DNA methy-lation level. This is particularly striking for Dppa5a and Spp1, but likely also for the other two (Fig. 3b). Depending on whether my interpretation is correct, this might be either (a) major concern with the appropriateness of the model requiring further improvement, convincing data / analysis, or removing the ”critical” claim from the paper, or (b) a minor concern that could be resolved my clarifying the misunderstanding (e.g., if the authors just mean to say ”not confounded by mean DNA methylation levels” as opposed to ”differences in mean methylation”).

*We apologise for the confusion. Indeed, the “differences” should not be present in that sentence and we have updated the manuscript accordingly. However, we want to emphasise that the changes in variability inferred by scMET (based on residual overdispersion estimates) are not associated to changes in mean methylation. This is illustrated in Figure R16 using the mouse brain cortex dataset.*

*Regarding the example genes shown in Figure 4 (such as Dppa5a and Spp1) we want to clarify that we did not perform any differential analysis across developmental stages. Instead, we applied scMET to quantify residual overdispersion across all cells. The examples shown in Figure 4b illustrate the behaviour of features with high DNAm variability, focusing on those with low (Krt8, Id3) or high (Dppa5a, Spp1) transcriptional variability (see Figure 4a). Cells were subsequently grouped by stage in Figure 4b, but only for visualisation purposes. These was done to highlight the developmental role observed for some of the examples. For clarity, Figure R17 shows the distribution of methylation values when considering all cells as a single group.*

*Figure R16. For each feature, the change in mean methylation across is plotted against the change in residual overdispersion between excitatory and inhibitory neurons. Features with statistically significant changes in vari-ability are coloured according to their regulation. Red-like and green-like colours denote features with higher variability in excitatory and inhibitory neurons, respectively.*

*Figure R17. Violin plots showing the distribution of methylation values across all cells for the Dppa5a and Spp1 genes.*

*Minor comments*

*1.	The term ”residual overdispersion” is quite important for the paper (first mention, l. 31), yet may not be common knowledge for many readers. Further (high-level) explanation would be beneficial. Perhaps, the first paragraph of the Methods should be included in the main text (introduction?).*

*We thank the reviewer for the suggestion to making our manuscript more accessible. We have now moved the first paragraph of the Methods section to the first sub-section of the Results.*

*2.	We are told scMET uses a Beta Binomial model, but it is not clear whether this model was chosen among other options for a particular reason or under some specific assumptions about the data (p. 3). Again, the meaning of this term should be briefly explained and a justification given (is it common knowledge [reference??]; is it empirical? Based on some prior model selection on the distributions of real datasets?).*

*To further motivate the choice of a beta-binomial (BB) model, we included a brief overview in Supplementary Note S2.1 (also highlighted in Methods). This is reproduced below.*

*Choice of a beta-binomial (BB) likelihood*

*The BB model has been successfully applied in the context of bulk DNA methylation data [13]; therefore constitutes a natural building block when modelling single cell DNA methylation measure¬ments. Here, we provide a brief description to motivate its use.*

*In a single-cell bisulphite experiment, the output is a binary readout (methylated or unmethylated) for each cell i and CpG site j. This can be modelled with a Bernoulli distribution. However, it is often preferred to pool information across CpG sites and instead work with predefined regions of several base pairs long (e.g. sliding windows, promoters or enhancers). Here, we refer to these regions as features. For each feature j and cell i, the number of methylated CpGs (Yij) can be modelled as:*

*Yijθj ∼ Binomial(nij, µj),*

*where ij represent the coverage for each cell and region. Within a region, this assumes that CpGs are independent and have the same probability of being methylated (j), which is constant across all cells. However, single cell methylation data typically exhibits more variability than what is predicted by this model. This is due to the presence of biological variability, e.g. multiple cell types in the population. This additional variability can be captured using the BB model, defined as:*

*ijij ∼ Binomial(ijij)	(2)*

*ijjj ∼ Beta(jj)	(3)*

*where j is an overdispersion parameter that ranges from 0 to 1. If j = 0 (no biological variability), this model reduces to the one in equation (1).*

3.	In defining the parameters of the model, the authors need to be very careful about the wording as this can be easily confusing readers not intimate to these data. In my experience, even most ’genomics experts’ come in their thinking from a perspective of transcriptomics, where it’s all about counting reads, and fail to immediately grasp the essentially binary nature of DNA methylation and the fact that there’s a limited number of DNA fragments that could have ever been measured in a single cell. ’For each cell i and feature j, the input for scMET is the number of CpG sites that are observed to be methylated (Yij ) and the total number of sites for which methylation status was recorded (nij )’ - what the authors mean by ’observed to be methylated’ and ’total [...] recorded’ is the number of CpG sites that were covered by a read (as opposed to the many NAs that were not covered at all). Those CpGs that were covered can either be methylated or not. I’d urge the authors to carefully consider / optimize their explanations here. Perhaps some of Figure 1b could be better used to illustrate this.

*Following reviewer’s suggestion, we have updated Figure 1b, to include example CpG sites where we have missing information and have modified the associated caption to more clearly state the nature of information we obtain from scBS-seq data. Additionally, we now provide a practical demonstration for the definition of ij andij (see Supplementary Fig. S1).*

4.	p.2, l. 24: ”To overcome sparsity, scMET aggregates the input data within regions (hereafter also referred to as genomic features): either by combining CpG information in a sliding window approach or using pre-annotated contexts”. Details should be given about the sliding window approach (which window sizes? Any restrictions? Does it matter?), and the analysis in Fig. 3 and 4 be performed also with these sliding windows (in addition to the pre-defined features).

*Following both reviewers’ suggestions, we extensively demonstrate the performance of scMET using sliding windows (see reply to point 1 from first Reviewer). We also added a section in Methods to describe the pipeline on how users should run scMET for genome-wide analysis.*

5.	The authors state that scMET is being prepared for submission to Bioconductor. That would be much appreciated. Can the dependency on v8 be dropped? I don’t understand why a Javascript engine would be required for scMET and the dependency makes installation more troublesome (because most users won’t have the system-level v8 dependency pre-installed).

*We agree with the reviewer that the dependency on V8 is not required, and indeed scMET does not directly import or depend on the V8 library. However, this occurs due to the dependency on the Rstan package, for which the new versions require the V8 library (as per this issue: https://github.com/stan-dev/rstan/issues/831). We have updated our Github repository with information on how the users without V8 can install scMET. See https://github.com/andreaskapou/scMET.*

*References*

*[1]	Luo, C., Keown, C. L., Kurihara, L., Zhou, J., He, Y., Li, J., ... & Ecker, J. R. (2017). Single-cell methylomes identify neuronal subtypes and regulatory elements in mammalian cortex. Science, 357(6351), 600-604.*

*[2]	Bardenet, R., Doucet, A., & Holmes, C. C. (2017). On Markov chain Monte Carlo methods for tall data. Journal of Machine Learning Research, 18(47).*

*[3]	Benton, M. L., Talipineni, S. C., Kostka, D., & Capra, J. A. (2019). Genome-wide enhancer annotations differ significantly in genomic distribution, evolution, and function. BMC Genomics, 20(1), 1-22.*

*[4]	Hui, T., Cao, Q., Wegrzyn-Woltosz, J., O’Neill, K., Hammond, C. A., Knapp, D. J., ... & Hirst, M. (2018). High-resolution single-cell DNA methylation measurements reveal epigenetically distinct hematopoietic stem cell subpopulations. Stem cell reports, 11(2), 578-592.*

*[5]	Juhling, F., Kretzmer, H., Bernhart, S. H., Otto, C., Stadler, P. F., & Hoffmann, S. (2016). Metilene: fast and sensitive calling of differentially methylated regions from bisulfite sequencing data. Genome Research, 26(2), 256-262.*

*[6]	Angermueller, C., Clark, S. J., Lee, H. J., Macaulay, I. C., Teng, M. J., Hu, T. X., ... & Reik, W. (2016). Parallel single-cell sequencing links transcriptional and epigenetic heterogeneity. Nature methods, 13(3), 229-232.*

*[7]	Clark, S. J., Argelaguet, R., Kapourani, C. A., Stubbs, T. M., Lee, H. J., Alda-Catalinas, C., ... & Reik, W. (2018). scNMT-seq enables joint profiling of chromatin accessibility DNA methylation and transcription in single cells. Nature communications, 9(1), 1-9.*

*[8]	Argelaguet, R., Clark, S. J., Mohammed, H., Stapel, L. C., Krueger, C., Kapourani, C. A., ... & Reik, W. (2019). Multi-omics profiling of mouse gastrulation at single-cell resolution. Nature, 576(7787), 487-491.*

*[9]	Kapourani, C. A., & Sanguinetti, G. (2019). Melissa: Bayesian clustering and imputation of single-cell methylomes. Genome Biology, 20(1), 1-15.*

*[10]	Kapourani, C. A., & Sanguinetti, G. (2018). BPRMeth: a flexible Bioconductor package for modelling methylation profiles. Bioinformatics, 34(14), 2485-2486.*

*[11]	Hansen, K. D., Langmead, B., & Irizarry, R. A. (2012). BSmooth: from whole genome bisulfite sequencing reads to differentially methylated regions. Genome biology, 13(10), 1-10.*

*[12]	Phipson, B., & Oshlack, A. (2014). DiffVar: a new method for detecting differential variability with application to methylation in cancer and aging. Genome biology, 15(9), 1-16.*

*[13]	Feng, H., Conneely, K. N., & Wu, H. (2014). A Bayesian hierarchical model to detect differentially methylated loci from single nucleotide resolution sequencing data. Nucleic acids research, 42(8), e69-e69.*

**Second round of review**

**Reviewer 1**

The authors have made substantial modifications to the manuscript which now fully addresses my questions. The revised manuscript now includes more performance comparisons, which illustrates the utility of the method,. I believe scMET will be a useful tool and manuscript is acceptable for publication in Genome Biology

**Reviewer 2**

I thank the authors for their thoughtful responses and believe that my concerns have been addressed sufficiently well. I recommend publication and only have a few, optional suggestions relating to figures:

Fig. 1d: Text in heading seems to be cut off at the bottom.

Fig. 1c,d,e, 2c,d, 3b,c,d: I would recommend always spelling out “DNA methylation” (instead of just “methylation”). Also the unit of these DNA methylation values may not be entirely clear – I would use 0…100% instead of 0…1, or add the word “ratio”.

Fig. 2b: Colors might be difficult to distinguish for color-blind readers.

Fig. 3b: Doubtful choice of color scale – I see only green vs. yellow. Values in between are indistinguishable.

Fig. 3a,c,d: Some very small text (“N=5242”, tick marks, etc.)
